# MicroRNAs as Bile-based biomarkers in pancreaticobiliary cancers (MIRABILE): a cohort study

**DOI:** 10.1097/JS9.0000000000001888

**Published:** 2024-07-23

**Authors:** Daniel S.K. Liu, Jisce R. Puik, Morten T. Venø, Mireia Mato Prado, Eleanor Rees, Bhavik Y. Patel, Nabeel Merali, Daniel Galloway, Grace Chan, Natalie Phillips, Christopher Wadsworth, Panagiotis Vlavianos, Jonathan Potts, Shivan Sivakumar, Brian R. Davidson, Marc G. Besselink, Rutger-Jan Swijnenburg, Long R. Jiao, Geert Kazemier, Elisa Giovannetti, Jonathan Krell, Adam E. Frampton

**Affiliations:** a Division of Cancer, Department of Surgery and Cancer, Imperial College London, Hammersmith Hospital, London, UK; b Department of Surgery, Amsterdam UMC Location Vrije Universiteit Amsterdam; c Cancer Center Amsterdam, Imaging and Biomarkers, Amsterdam, The Netherlands; d Department of Molecular Biology and Genetics, Interdisciplinary Nanoscience Center, Aarhus University, Aarhus C; e Omiics ApS, Aarhus N, Aarhus, Denmark; f Department of Clinical and Experimental Medicine, Faculty of Health and Medical Sciences, Section of Oncology, The Leggett Building, University of Surrey; g HPB Surgical Unit, Royal Surrey NHS Foundation Trust, Guildford, Surrey; h Department of Gastroenterology, Chelsea and Westminster Hospital, Chelsea and Westminster Hospital NHS Foundation Trust, London; i Department of Gastroenterology, Hammersmith Hospital, Imperial College Healthcare NHS Trust, Du Cane Road, London, W12 0HS; j Royal Free Sheila Sherlock Liver Centre, Royal Free Hospital and UCL Institute of Liver and Digestive Health, London; k Department of Oncology, Institute of Immunology and Immunotherapy, Birmingham Medical School, University of Birmingham, Birmingham; l Department of HPB and Liver Transplant Surgery, Royal Free Hospital; m Division of Surgery and Interventional Science, Faculty of Medical Sciences, University College London, London, UK; n Department of Surgery, Amsterdam UMC location University of Amsterdam, Meibergdreef, Amsterdam, The Netherlands; o Department of Surgery and Oncology, The Royal Marsden Hospital, London, UK; p Cancer Pharmacology Lab, Fondazione Pisana per la Scienza, Pisa, Italy

**Keywords:** bile, biomarker, cholangiocarcinoma, microRNA, pancreatic ductal adenocarcinoma

## Abstract

**Background:**

Biliary obstruction can be due to both malignant and benign pancreaticobiliary disease. Currently, there are no biomarkers that can accurately help make this distinction. MicroRNAs (miRNAs) are stable molecules in tissue and biofluids that are commonly deregulated in cancer. The MIRABILE study aimed to identify miRNAs in bile that can differentiate malignant from benign pancreaticobiliary disease.

**Materials and methods:**

There were 111 patients recruited prospectively at endoscopic retrograde cholangiopancreatography (ERCP) or percutaneous transhepatic cholangiography (PTC) for obstructive jaundice, and bile was aspirated for cell-free RNA (cfRNA) extraction and analysis. In a discovery cohort of 78 patients (27 with pancreatic ductal adenocarcinoma (PDAC), 14 cholangiocarcinoma (CCA), 37 benign disease), cfRNA was subjected to small-RNA sequencing. LASSO regression was used to define bile miRNA signatures, and NormFinder to identify endogenous controls. In a second cohort of 87 patients (34 PDAC, 14 CCA, 39 benign disease), RT-qPCR was used for validation.

**Results:**

LASSO regression identified 14 differentially-expressed bile miRNAs of which 6 were selected for validation. When comparing malignant and benign pancreaticobiliary disease, bile miR-340 and miR-182 were validated and significantly differentially expressed (*P*<0.05 and *P*<0.001, respectively). This generated an AUC of 0.79 (95% CI: 0.70–0.88, sensitivity 65%; specificity 82%) in predicting malignant disease.

**Conclusion:**

Bile collected during biliary drainage contains miRNAs able to differentiate benign from malignant pancreaticobiliary diseases in patients with obstructive jaundice. These bile miRNAs have the potential to increase diagnostic accuracy.

## Graphical abstract

**Figure:**
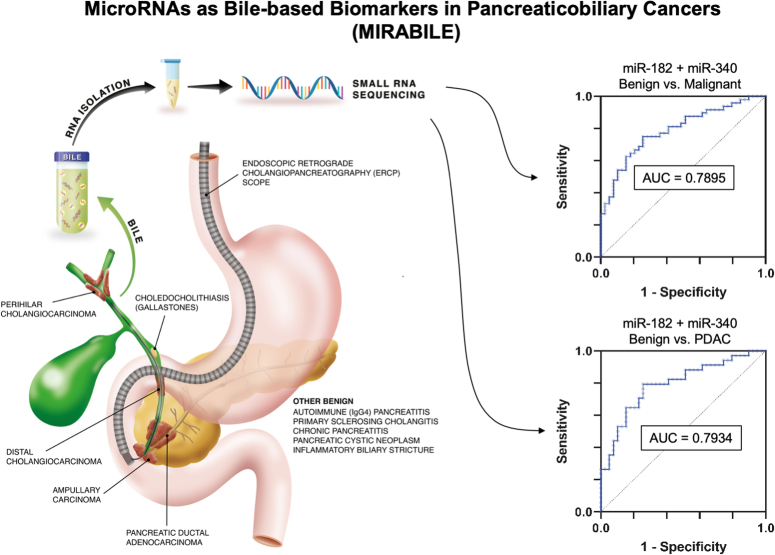

## Introduction

Highlights

- This is the first study to assess bile miRNAs by small RNA-sequencing in a large number of patients presenting with obstructive jaundice.
- This study demonstrated that bile cell-free miRNAs can be a source of biomarkers during a ‘window of opportunity’ prior to anticancer treatment to help stratify and personalise patient pathways.

Obstructive jaundice can be a clinical sign indicating occlusion or narrowing of bile ducts due to benign or malignant pancreaticobiliary disease. The most common malignant cause is pancreatic ductal adenocarcinoma (PDAC)^1^. Other malignant causes include cholangiocarcinoma (CCA) and ampullary cancer. Accurate diagnosis is important to prevent futile surgical resection in case of benign disease, such as chronic pancreatitis, and to treat malignant disease in a timely manner and avoid the chance of disease progression. However, obstructive jaundice with secondary biliary inflammation can make it challenging to recognise distinctive radiological features, or to obtain representative samples during endoscopic sampling^1,2^. Up to 20% of the biliary strictures may remain indeterminate, raising a therapeutic dilemma that is often decided by the maxim that a biliary stricture is malignant until proven otherwise^3^. Even when a biliary stricture is suspected malignant, it has been reported that 10% of these patients who undergo resection are postoperatively diagnosed with benign conditions^3–5^. To improve the diagnosis of biliary strictures causing obstructive jaundice, the MIRABILE study was designed to assess the feasibility and diagnostic value of cell-free RNAs (cfRNAs) in bile as diagnostic biomarkers to differentiate malignant from benign lesions.

Diagnostic work-up for a biliary stricture includes laboratory tests, imaging and endoscopic modalities. The diagnostic efficacy of blood-based biomarker carbohydrate antigen 19-9 (CA 19-9) has previously been assessed, but has been shown inadequate as a stand-alone biomarker to discriminate PDAC from benign pancreaticobiliary disease^6^. Serum CA 19-9 has a respective sensitivity and specificity of 78.2 and 82.8% when the threshold is set at 40 U/ml^6^. This is similar to computed tomography (CT), which has a respective sensitivity and specificity of 77% and 63% in the recognition of malignant versus benign biliary obstruction^7^. Contrast-enhanced MRI with magnetic resonance cholangiopancreatography (MRCP) has shown a sensitivity and specificity of ~85% for detecting malignant biliary strictures^8–11^. Furthermore, endoscopic retrograde cholangiopancreatography (ERCP) with brush cytology has a pooled sensitivity of 41–45%, while this is 48% for intraductal biopsy^2,12^. Endoscopic procedures remain the most optimal route to make a cytological diagnosis, enabling tissue or cells to be obtained. Exploring biomarkers from bile aspirated at this stage offers two benefits. Firstly, it is one of the initial stages in the patient’s diagnostic journey to manage biliary obstruction^1,3^. Secondly, bile is intimately related to the tumour site in terms proximity and anatomy, which enhances the potential for detecting molecular markers for pancreaticobiliary cancers^13–16^.

CfRNAs can be assessed as molecular markers in many bodily fluids, including blood^17^ and bile^18^. Of these cfRNAs, extracellular microRNAs (miRNAs) appear to be selectively protected from endogenous RNases found within the blood^19^. Furthermore, they are stable through freeze-thaw cycles and extremes of pH^20,21^. MiRNAs have a known role in the regulation of gene expression at a post-transcriptional level and are involved in many biological processes. They have shown unique expression profiles in tumours and have hitherto been shown to be differentially expressed markers in numerous biofluids^22^. MiRNAs reflect the current state of cells and tissues and, as such, form an attractive class of biomarkers for diagnostic purposes^23,24^.

MIRABILE (MIcroRNAs As BILE-based biomarkers in Pancreaticobiliary Cancers) was a prospective study that assessed cfRNAs in bile obtained from patients with pancreaticobiliary disease who presented at ERCP for obstructive jaundice. We identified a novel bile-based miRNA signature for the diagnosis of malignant pancreaticobiliary disease.

## Materials and methods

### Study design

**Figure 1:**
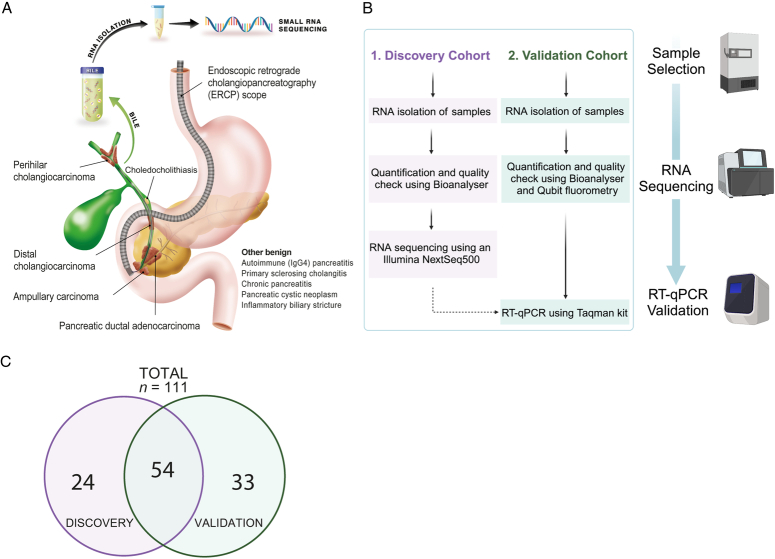
Study design of the MIRABILE study. (A) A graphical figure demonstrating the overall study design together with relevant pancreaticobiliary diseases. (B) Schematic diagram showing the workflow: RNA was isolated from bile samples, followed by quantification and then either RNA sequencing analysis or RT-qPCR. (C) Venn diagram showing the distribution of patients between the discovery and validation cohorts used in the analysis of bile cfRNA. RT-qPCR, quantitative reverse transcription polymerase chain reaction.

All study-related procedures and protocols were in accordance with the ethical guidelines of the Declaration of Helsinki and local ethical committees. Written informed consent was obtained from all individual subjects. The study is reported according to the Standars for the Reporting of Diagnostic accuracy studies (Supplemental Digital Content (SDC) 1, http://links.lww.com/JS9/D148, Figure S1; SDC 2, STARD Checklist, Supplemental Digital Content 2, http://links.lww.com/JS9/D149)^25^. We collected and analysed 111 bile samples from patients with benign and malignant pancreaticobiliary disease in this two-phase study (Fig. 1A-B), which consisted of biomarker discovery by small RNA sequencing and validation by RT-qPCR. Bile samples were allocated to the discovery or validation cohort based on sample availability Figure 1C.

### Sample collection and preparation

Bile was collected prospectively from consecutive patients attending the endoscopy unit between 2nd November 2017 and 11th March 2020. All patients were attending the endoscopic unit for evaluation of biliary obstruction. Inclusion criteria for patients recruited were age ≥18 years, able to provide written informed consent, scheduled for clinical reason to undergo an ERCP or a PTC, and WHO performance status <3. Exclusion criteria included patients with previous Roux-en-Y gastrojejunostomy, hepaticojejunostomy or Billroth II, pregnancy or those with WHO performance status 3 or 4. Definitions of benign and malignant, as well as procedures of sample collection and data collection are described in SDC 3, Materials and Methods (Supplemental Digital Content 3, http://links.lww.com/JS9/D150).

### RNA isolation, analysis, library preparation, and quantification

Full detailed protocols for RNA isolation, library preparation and quantification are described in SDC 3, Materials and Methods (Supplemental Digital Content 3, http://links.lww.com/JS9/D150). Total RNA was isolated using TRIzol LS Reagent (Thermo Fisher Scientific) from 500 µl bile. RNA samples were prepared for sequencing using QIAseq small RNA Library Prep kit (Qiagen) and sequenced on a NextSeq 500 system (Illumina). Validation of candidate miRNAs was performed using Reverse Transcription Quantitative Polymerase Chain Reaction (RT-qPCR) and target-specific stem–loop primer assays. C_T_ values were calculated and normalised to endogenous miRNAs.

### Statistical analysis

Small RNA-sequencing was used to identify differentially expressed miRNAs in bile. An adjusted *P*<0.05 was considered statistically significant. Receiver operating characteristic (ROC) curve analyses were performed for significantly upregulated miRNAs that showed the highest fold change (minimum log2(fold change) >2), leading to estimates of the area under the curve (AUC) with 95% CI. To prevent selection bias, all statistically significant miRNAs were included in Least Absolute Shrinkage and Selection Operator (LASSO) regression analysis. This type of regression analysis was chosen and performed in order to identify a parsimonious model^26^. It is a computational approach that uses L1 regularisation to combine potential candidates and reduce coefficients from a wide number of miRNA candidates to zero (i.e. adds penalty equivalent to the absolute value of the magnitude of coefficients)^26^. A selection of upregulated miRNAs (i.e. those that were considered upregulated and expressed in cancer may be more pragmatic to apply as cancer biomarker) was validated by RT-qPCR. Since strong collinearity between input variables (i.e. microRNAs) for logistic regression influences the accurate estimation of the miRNA model, Spearman’s Rank correlation analysis was performed for miRNAs and several laboratory markers. Next, multiple logistic regression analyses were performed to generate ROCs for potential diagnostic miRNAs and determine corresponding AUC values. Optimum cut-offs for sensitivity and specificity were determined from the ROC curve at the maximum Youden index.

### MicroRNA functional analysis

To identify potential functional targets for the candidate miRNAs, gene set enrichment analysis (GSEA) tailored to miRNA was used to evaluate pathways, Gene Ontology terms, and targets^27^. Analyses were performed using the web-based application miRNA Enrichment Analysis and Annotation tool 2.1 (miEAA2.1). The input list of miRNAs was sorted in order of *P*-value from validation data. Validated miRNA-target interactions were obtained from miRWalk 2.0, HMDD2 and miRTarbase and converted to the current miRBase v21. *P*-values were computed by applying the Fisher’s exact test and adjusted for multiple comparisons (Bonferroni) with a standard significance threshold of *P*<0.05.

## Results

### Patients

Patient details are summarised in Table 1 and further described in SDC 4, Supplementary Results (Supplemental Digital Content 4, http://links.lww.com/JS9/D151). A total of 111 samples were collected prospectively from patients presenting for biliary drainage. A total of 78 samples (27 PDAC, 14 CCA, and 37 benign) were selected for the discovery cohort. The validation cohort comprised 87 samples (34 PDAC, 14 CCA, and 39 benign).

**Table 1:** Clinicopathological characteristics of patients in the discovery and validation cohorts.

	Total (*n*=111)				Discovery (*n*=78)				Validation (*n*=87)			
	PDAC (*n*=38)	CCA (*n*=19)	Benign (*n*=54)	*P*	PDAC (*n*=27)	CCA (*n*=14)	Benign (*n*=37)	*P*	PDAC (*n*=34)	CCA (*n*=14)	Benign (*n*=39)	*P*
Age, years												
<60	8 (21)	4 (21)	24 (44)		8 (30)	3 (21)	16 (43)		6 (18)	3 (21)	18 (46)	
>60	30 (79)	15 (79)	30 (56)	**0.0143**	19 (70)	11 (79)	21 (57)	0.1565	28 (82)	11 (79)	21 (54)	**0.0098**
Mean (SD)	69 (11)	68 (9)	62 (16)	0.0054	68 (11)	65 (7.8)	63 (16)	0.1260	70 (11)	70 (8)	61 (16)	**0.0029**
Sex (%)												
Female	13 (34)	6 (32)	21 (39)		12 (44)	4 (29)	15 (41)		11 (32)	4 (29)	14 (36)	
Male	25 (66)	13 (68)	33 (61)	0.5596	15 (56)	10 (71)	22 (59)	0.8254	23 (68)	10 (71)	25 (64)	0.6557
History of PSC (%)	0 (0)	1 (5)	4 (7)	0.2017	0 (0)	0 (0)	3 (8)	0.1105	0 (0)	1 (7)	4 (10)	0.1688
Chronic Pancreatitis (%)	4 (11)	0 (0)	11 (20)	0.0540	3 (11)	0 (0)	7 (19)	0.1858	4 (12)	0 (0)	9 (23)	0.0723
Blood parameters												
CA19-9 (U/ml)	9341	3186	278	0.0770	7825	555	347	0.1023	10158	4298	287	0.0823
Bilirubin (ng/ml)	248	148	42	**<0.0001**	244	110	46	**<0.0001**	254	149	48	**<0.0001**
CRP (μg/ml)	50	57	48	0.9643	49	58	53	0.4931	52	47	38	0.7892
Anatomical location												
Perihilar CCA	—	10 (53)	—		—	9 (64)	—		—	7 (50)	—	
Distal CCA	—	7 (37)	—		—	5 (36)	—		—	5 (36)	—	
Intrahepatic CCA	—	2 (11)	—		—	0 (0)	—		—	2 (14)	—	
Head of Pancreas PDAC	31 (82)	—	—		21 (78)	—	—		27 (79)	—	—	
Uncinate PDAC	3 (8)	—	—		3 (11)	—	—		3 (9)	—	—	
Mixed PDAC (HOP/uncinated)	4 (11)	—	—		3 (11)	—	—		4 (12)	—	—	
Tumour stage* (%)												
T1	0 (0)	0 (0)	—		0 (0)	0 (0)	—		0 (0)	0 (0)	—	
T2	7 (18)	3 (17)	—		6 (22)	3 (21)	—		7 (21)	1 (7)	—	
T3	10 (26)	10 (53)	—		7 (26)	8 (58)	—		8 (24)	7 (50)	—	
T4	19 (50)	6 (33)	—		14 (52)	3 (21)	—		19 (56)	6 (43)	—	
Nodal disease (%)	23 (61)	8 (42)	—		18 (66)	6 (42)	—		23 (68)	5 (36)	—	
Metastatic disease (%)	11 (29)	1 (6)	—		6 (22)	0 (0)	—		11 (32)	1 (7)	—	

*In accordance with the eight edition AJCC Cancer Staging Manual.

*P*-values <0.05 are highlighted in bold.

CA19-9, carbohydrate antigen 19-9; CCA, cholangiocarcinoma; CRP, C-reactive protein; HOP, head of pancreas; PDAC, pancreatic ductal adenocarcinoma.

### Small RNA sequencing of bile cfRNA revealed differentially expressed bile miRNAs

**Figure 2:**
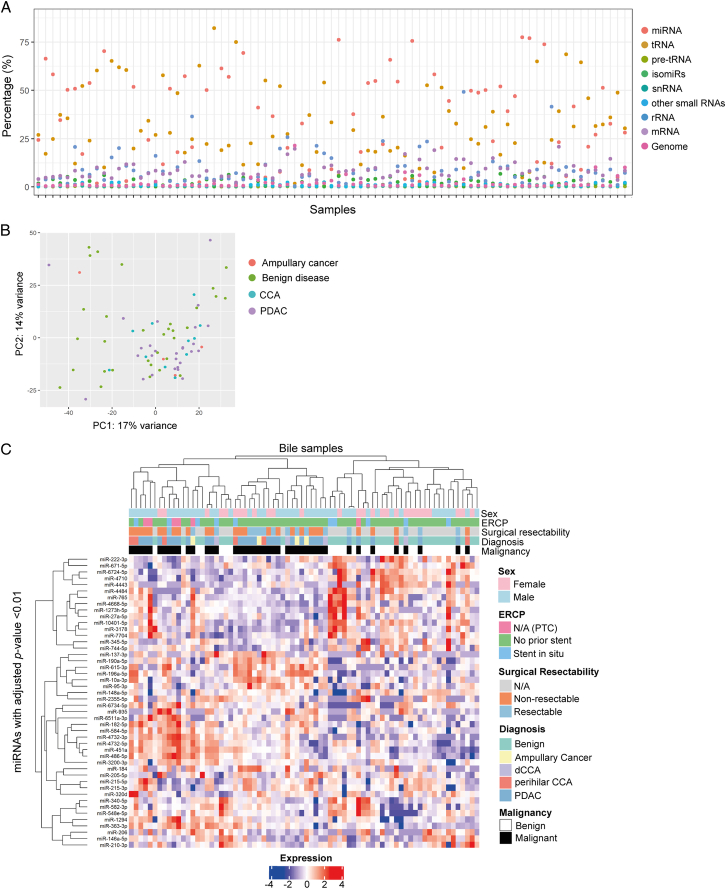
Bile cell-free RNA (cfRNA) demonstrated differences in RNA quantity and expression. (A) Relative distribution of small RNA reads for each bile sample from the discovery cohort (*n*=78). (B) Principal component analysis (PCA) plot of bile miRNA expression showing clustering of samples. (C) Heatmap with unsupervised hierarchical clustering showing malignant samples grouping to the left and including all miRNAs with adjusted *P*-values <0.01. CCA, cholangiocarcinoma; dCCA, distal CCA; ERCP, endoscopic retrograde cholangiopancreatography; mRNA, messenger RNA; N/A, not applicable; PC, principal component; PDAC, pancreatic ductal adenocarcinoma; PTC, percutaneous transhepatic cholangiography; rRNA, ribosomal RNA; snRNA, small nuclear RNA; tRNA, transfer RNA.

The composition of small noncoding RNAs that were present in bile samples of the discovery cohort is shown in Figure 2A. MiRNAs were a highly expressed species of RNA in human bile (mean proportion of reads was 36%). After filtering for reads, a total of 457 miRNAs were identified for differential expression analysis. Correlation of different samples according to miRNA expression values is shown in a principal component analysis (PCA) plot (Fig. 2B). For miRNAs with adjusted *P*-values <0.01, a heatmap was generated with unsupervised hierarchical clustering according to similar expression in samples (Fig. 2C).

**Figure 3:**
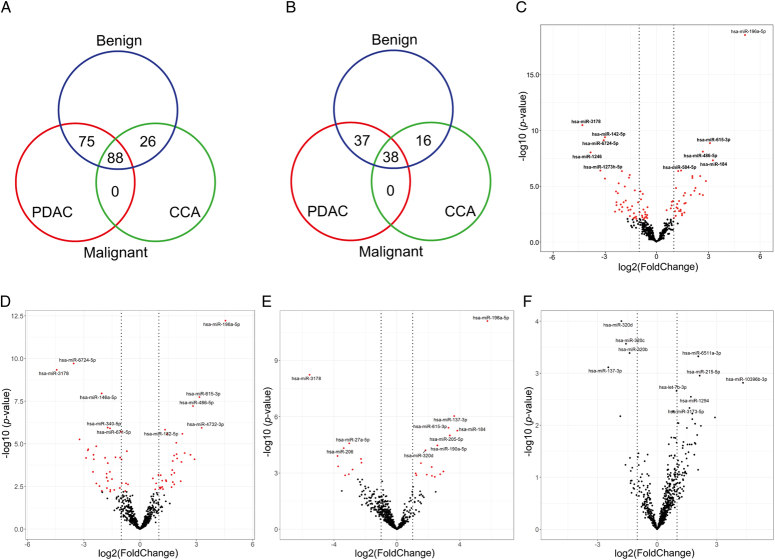
Differential expression analysis identified differentially expressed miRNAs in bile samples. Venn diagrams for (A) the total number of differentially expressed miRNAs (adjusted *P-*value <0.05), and (B) the total number of upregulated miRNAs only when comparing PDAC, CCA, and benign cohorts. Volcano plots demonstrate differentially expressed miRNAs with the top 10 most statistically significant miRNAs annotated (upregulated and downregulated) for the pairwise comparisons: (C) malignant (PDAC and CCA) vs. benign disease; (D) PDAC vs. benign; (E) CCA vs. benign and (F) PDAC vs. CCA. Red illustrates miRNAs with FDR<0.05. Vertical dotted lines indicate log2(fold change)=+/− 1. CCA, cholangiocarcinoma; hsa, homo sapiens; PDAC, pancreatic ductal adenocarcinoma.

The numbers of statistically significantly (adjusted *P*<0.05) differentially expressed miRNAs for all pairwise comparisons are displayed in a Venn diagram in Figure 3A. From these dysregulated miRNAs, upregulated miRNAs may be to more pragmatic as clinical diagnostic biomarkers (i.e. considered upregulated and identifiable in samples). Upregulated miRNAs are hence shown in a second Venn diagram (Fig. 3B). When comparing malignant and benign disease, 88 miRNAs were significantly differentially expressed (Fig. 3C). The top 20 dysregulated miRNAs with highest fold change are shown in SDC 5, Table S1 (Supplemental Digital Content 5, http://links.lww.com/JS9/D152). Furthermore, 75 miRNAs showed differential expression for the pairwise comparison PDAC vs. benign disease (Fig. 3D; SDC 5, Table S2, Supplemental Digital Content 5, http://links.lww.com/JS9/D152), 26 for CCA vs. benign disease (Fig. 3E; SDC 5, Table S3, Supplemental Digital Content 5, http://links.lww.com/JS9/D152), and 0 for CCA vs. PDAC (Fig. 3F). Although analysis of transfer RNA fragment (tRF) expression was not the aim of this study, small RNA sequencing revealed that tRFs were highly expressed in our bile cfRNA samples and on average made up 37% of the small RNA composition in bile (Fig. 2A). In order to evaluate these for future investigations, differential expression analysis was performed using the available small RNA sequencing data (SDC 4, Supplementary Results, Supplementary Results, Supplemental Digital Content 4, http://links.lww.com/JS9/D151).

### Validation of candidate miRNAs from LASSO regression analysis by RT-qPCR

All miRNAs with adjusted *P*<0.05 were used as input for LASSO regression analysis. Further details and results of LASSO regression analysis are described in SDC 4, Supplementary Results, Supplementary Results (Supplemental Digital Content 4, http://links.lww.com/JS9/D151). Following LASSO regression analysis, six upregulated miRNAs (i.e. those that were considered upregulated and expressed in cancer may be easier to apply as cancer biomarker) were selected as candidates for RT-qPCR validation: miR-196a-5p, miR-199a-5p, miR-335-5p, miR-182-5p, miR-340-5p and miR-424-5p. These six miRNAs were selected because a) they were all included in the 14-miRNA LASSO model that discriminated malignant from benign disease with an AUC of 0.93 (95% CI: 0.84–1.00), and b) the first four miRNAs were also included in the LASSO regression model that discriminated PDAC from benign disease. Endogenous miRNAs with stable expression were used to calculate the relative expression of candidate miRNAs. These normalisers were identified by applying the NormFinder algorithm to small RNA sequencing data (SDC 5, Table S4, Supplemental Digital Content 5, http://links.lww.com/JS9/D152)^28^. As a result, miR-26a and miR-let-7b were used as normalisers in subsequent RT-qPCR analyses.

During validation, two of the six miRNAs (miR-199a-5p and miR-424-5p) did not show adequate expression (>50% of samples showing no expression) and were not further assessed. Expression levels of the other four candidate miRNAs (miR-196a-5p, miR-182-5p, miR-335-5p, and miR-340-5p) were considered adequate for further analysis. Correlation analysis of these miRNAs is described in SDC 4, Supplementary Results, Supplementary Results (Supplemental Digital Content 4, http://links.lww.com/JS9/D151).

**Figure 4:**
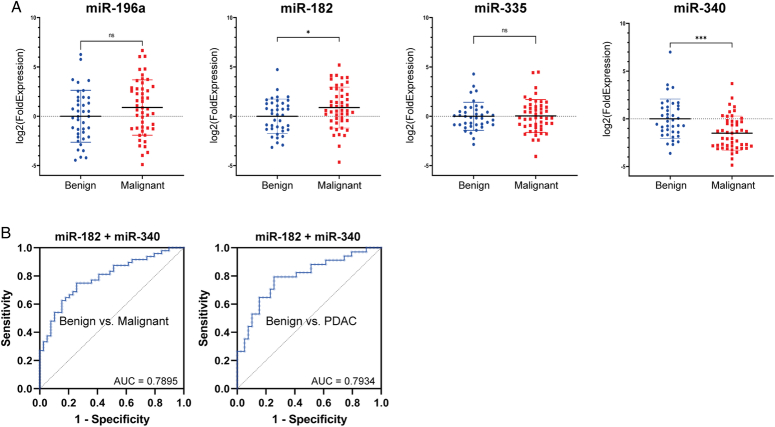
RT-qPCR validation of candidate miRNAs identified by LASSO regression analysis. (A) Expression of candidate miRNAs in benign and malignant pancreaticobiliary disease (left to right): miR-196a, miR-182, miR-335 and miR-340. *P<0.05, **P<0.005, ***P<0.0005. (B) ROC curve with corresponding AUC value for the 2-miRNA signature (miR-182 and miR-340) to predict malignant disease vs. benign disease (left), or to predict PDAC vs. benign disease (right). AUC, area under the curve; PDAC, pancreatic ductal adenocarcinoma.

Differential expression of the four final candidate miRNAs were evaluated in the pairwise comparison of malignant (i.e. PDAC and CCA) vs. benign disease is shown in Figure 4A. MiR-182-5p was significantly upregulated in malignant samples (*P*=0.0179), while miR-340-5p was significantly downregulated in malignant samples (*P*=0.0002). MiR-196a-5p showed a trend towards upregulation in malignancy (*P*=0.0671) and miR-335-5p showed no significant difference between malignant and benign samples (*P*=0.4607). When PDAC and CCA were compared to benign disease individually, no statistically significant difference was shown for miR-182-5p, miR-196a-5p, and miR-335-5p. However, miR-340-5p was statistically significantly differentially expressed in both PDAC and CCA comparisons with benign disease (*P*=0.0275 and *P*=0.0007, respectively).

### A bile miRNA signature with diagnostic potential to discriminate malignant from benign disease

Within the validation cohort, serum CA 19-9 values were missing for 25 out of 39 (64%) patients with benign disease, 4 out of 48 (8%) patients with malignant disease, and 2 out of 34 (6%) patients with PDAC. Logistic regression removes patients with missing values for CA 19-9, resulting in a smaller sample size and potential bias in effect estimates. ROC and AUCs were calculated for CA 19-9 only (SDC 1, Figure S2, http://links.lww.com/JS9/D148), but in order to generate the most reliable miRNA signature CA 19-9 was not included in logistic regression analysis with candidate miRNAs.

Multiple logistic regression was performed for the miRNA combination miR-182-5p and miR-340-5p, generating a ROC curve which showed an AUC of 0.79 (95% CI: 0.70–0.88) in predicting malignant vs. benign disease (Fig. 4B, left). At the optimum cut-off, determined by maximum Youden Index, sensitivity and specificity were 64.6 and 82.1%, respectively. In addition, the negative predictive value (NPV) was 65.3% and the positive predictive value (PPV) 81.6%. In discriminating PDAC from benign disease, the miRNA signature showed an AUC of 0.79 (95% CI: 0.69–0.90) with a sensitivity of 82% and specificity of 59% (Fig. 4B, right). Moreover, NPV and PPV were 79 and 64%, respectively.

### Functional analysis of candidate miRNAs to identify common pathways

Fourty functional pathways were identified for all six candidates with an adjusted *P*-value <0.05. The top 10 is shown in SDC 5, Table S5 (Supplemental Digital Content 5, http://links.lww.com/JS9/D152). This analysis highlighted pathways including bone morphogenetic proteins (BMP) signalling, Toll-like receptor (TLR) pathways and involvement of the endosome. BMPs regulate developmental epithelial-to-mesenchymal transition (EMT) and there is evidence that there is a role for BMP signalling in promoting the metastatic cascade^29^.

## Discussion

Differential diagnosis between malignant and benign disease in patients with obstructive jaundice is challenging. As bile is in close proximity to the tumour and can be easily obtained during ERCP, we have identified bile miRNAs as compelling diagnostic biomarkers in the differentiation of patients with malignant and benign biliary strictures. This study is the largest study to date to identify miRNAs from bile using small RNA sequencing (see SDC 5, Table S6, Supplemental Digital Content 5, http://links.lww.com/JS9/D152, for an overview of previous studies on bile miRNAs). In total, 111 unique patient samples were used in this study, with a sequencing strategy to determine miRNA (and tRF candidates) from a discovery cohort of 78 samples (27 PDAC, 14 CCA, and 37 Benign). We were able to investigate all 2656 mature known human miRNAs (miRBase v22) and all 26 531 known tRNA fragments (MINTbase v2.0) in bile, indicating that bile is an ample source for potential RNA markers. A total of 457 miRNAs were identified with sufficient expression for analysis. After multiple hypotheses correction, 88 differentially expressed miRNAs were identified when comparing malignant and benign disease, 75 when comparing PDAC and benign disease and 26 when comparing CCA and benign disease. Using LASSO regression analysis, a sparse model was generated which included 14 miRNAs that could discriminate benign from malignant disease with an AUC of 0.933. Six miRNAs were chosen for validation RT-qPCR analysis, which confirmed a significant upregulation of miR-182-5p and downregulation of miR-340-5p in malignant samples. In combination, they showed an AUC value of 0.79 (95% CI: 0.70–0.88, *P*<0.0001) for predicting malignancy, with a sensitivity of 64.6% and of specificity 82.1%, and an AUC of 0.79 (95% CI: 0.69–0.90) for predicting PDAC, with a sensitivity of 82% and specificity of 59%. Compared to the CancerSEEK test, a blood test that assesses circulating proteins and mutations in cell-free DNA for early cancer detection, our miRNA signature showed a higher sensitivity for detecting PDAC (82 vs. 70%)^30^. A previous study already showed that miRNAs can also be assessed in the bile obtained from the gallbladder by surgical aspiration^18^. The study used nCounter Nanostring profiling to identify candidate miRNAs from surgically aspirated bile, and concluded that bile-based miR-148a-3p, miR-125b-5p, and miR-194-5p could be used to distinguish pancreaticobiliary disease. Compared to this previous study, we performed discovery by small RNA sequencing of a wider range of miRNAs (all known miRNAs according to miRbase v22, which includes 2656 mature miRNAs), and collected bile directly from the bile duct in a preoperative setting during ERCP. Therefore, we were able to obtain candidates that were different, but with greater predictive ability for pancreaticobiliary malignancy (AUC of 0.752 using miR-148a-3p compared with 0.796 using miR-340 and miR-182). No significantly differentially expressed miRs were identified comparing PDAC to CCA, which may be due to the small samples size for CCA (*n*=14), which contributes to the probability of a type II error.

Functional analysis of the candidate miRNAs indicated involvement in BMP and TLR pathways. Several BMP family members, in particular BMP 2 and 4, have been shown to induce EMT through increased matrix metalloproteinase (MMP)-2 and suppression of Smad1 in Panc-1 cells^29^. Furthermore, TLRs are known to be upregulated in PDAC and play a role in the antitumour immune response and inflammation through the recognition of pathogen and danger-associated molecular patterns^31^. Indeed, TGF-β induced miR-182 has been shown to modulate the TLR4/NF-κB signalling pathways, indicating its role in tumour progression^32,33^. Upregulation of our validated miR-182 has previously also been described in PDAC compared to chronic pancreatitis in blood, and in CCA compared to chronic cholangitis in cells from bile cytology^34,35^. Similarly, miR-340 has previously been shown to be downregulated in PDAC, and upregulation can be shown to inhibit tumour growth by influencing the tumour immune microenvironment in murine models^36^.

Surprisingly, small RNA sequencing also revealed that tRNA-derived fragments (tRFs) were highly expressed in bile and on average tRNAs composed 37% of the assessed bile samples. tRNA biology is complex with over 270 different tRNA sequences present among approximately 450 tRNA human genes^37^. It undergoes several chemical modifications which can alter its fragmentation and function^38^. Using high-throughput sequencing, several studies have reported on differential expression of tRFs in cancer, but also emphasised that further extensive investigations are warranted to understand the roles and underlying mechanisms of these RNA fragments^38^. Although this study focused on miRNAs, our preliminary findings may contribute to future studies into the potential roles of tRFs in pancreaticobiliary cancer.

Our study contained several limitations. Firstly, an independent validation in a separate cohort would provide stronger evidence for our model and would strengthen implementation in clinical practice. Secondly, a prediagnostic cohort of patients presenting with biliary obstruction was investigated, which meant that patients presented to our study with advanced disease. Furthermore, LASSO regression is a useful tool to identify biomarker signatures, but it does not take into account functional relationships. Correlation analysis did, however, show low correlation between candidate miRNAs and current laboratory markers, indicating a potential cancer-related role distinct from inflammation or obstruction. Our methodology also had a number of notable strengths. Firstly, we used an NGS approach for biomarker discovery. NGS is the preferred standard for miRNA biomarker discovery because it does not cause *a priori* selection bias^39^. Secondly, a relatively large cohort of patients was sequenced (*n*=78), after which results were also validated in a second cohort by RT-qPCR. Moreover, this is the first study to analyse tRFs in bile for diagnosis of PDAC and CCA^38^.

This study was designed for the hypothesis that bile-based microRNAs could act as an adjunct to help clinical decision making. Future studies could assess the diagnostic performance of a model including routinely measured biomarkers (e.g. CA 19-9 and bilirubin), bile-based miRNAs, and other novel markers (e.g. radiomics-base machine learning models, circulating tumour DNA, and microbiome)^40–44^. Furthermore, RNA sequencing in patients with PDAC has previously been used to develop promising tissue based transcriptomic predictive signatures for response to gemcitabine (*GemCore*) or FOLFIRINOX (single sample classifier PurIST)^45,46^. Possibly, bile-based miRNAs could contribute to such predictive signatures. Therefore, in our ongoing prospective multicentric clinical trial, RESECTA-BILE, we are studying bile miRNAs to validate their diagnostic potential, but also to investigate their potential roles as prognostic and predictive biomarkers for patients with resectable or borderline resectable PDAC.

## Conclusion

In conclusion, there is differential expression of miRNAs in bile associated with malignant pancreaticobiliary disease. This is the first study to assess bile miRNAs by small RNA-sequencing in a large number of patients presenting with obstructive jaundice. This study indicates that bile miRNAs could be clinically useful biomarkers for confirming malignant pancreaticobiliary disease. This opens the possibility that these bile miRNAs could be used during a ‘window of opportunity’ prior to anticancer treatment to help stratify and personalise patient pathways. The final diagnostic model with miR-182-5p and miR-340-5p generated an AUC of 0.79 for the detection of malignancy, and 0.79 when used for the prediction of PDAC only. Functional target analysis showed that these two miRNAs target BMP signalling, indicating that they could be associated with EMT and tumour progression. Thus, our findings demonstrated that bile cell-free miRNAs can be a source of biomarkers and potential adjuncts for the detection of PDAC and CCA.

## Ethical approval

All study-related procedures and protocols were in accordance with the ethical guidelines of the Declaration of Helsinki. Ethical approval was obtained as part of the Imperial College Healthcare Tissue Bank (ICHTB). ICHTB was approved on July 25th 2017 by Wales REC3 to release human material for research (ICHTB HTA licence 12275; REC Wales approval 17/WA/0161).

## Consent

Written informed consent was obtained from the patient for publication of this case report and accompanying images. A copy of the written consent is available for review by the Editor-in-Chief of this journal on request.

## Source of funding

This research was funded by The Jon Moulton Charity Trust, UK (AEF); Action Against Cancer, UK (DKSL, MMP, ER, BP, LRJ, JK AEF); the Royal College of Surgeons of Edinburgh, UK (AEF); the Royal College of Surgeons of England, UK (AEF, DSKL); the Mason Medical Research Foundation, UK (AEF); the S.A.L. Charitable Fund, UK (AEF); the Bennink Foundation, the Netherlands (JRP, GK, EG); Italian Association for Cancer Research AIRC, Italy (EG), Fondazione Pisana Per La Scienza, Italy (EG); and the Dutch Cancer Society KWF (RJS, EG, GK). These sponsors did not play a role in the collection, analysis or interpretation of data; nor in the writing of the manuscript; nor in the decision to submit the manuscript for publication.

## Author contribution

D.G., G.C., N.P., C.W., P.V., J.P., S.S., B.R.D., L.R.J., J.K., and A.E.F.: sample provision; D.S.K.L. and A.E.F.: clinical data collection; D.S.K.L., J.R.P., A.E.F., and E.G.: original draft preparation; A.E.F., J.K., E.G., and G.K.: funding acquisition. All authors contributed in concept and design, read and approved the final manuscript, and statistical analysis and interpretation, and critical revision and editing of the manuscript.

## Conflicts of interest disclosure

The authors have no disclosures for this work.

## Research registration unique identifying number (UIN)

The unique identifying number that was issued by ClinicalTrials.gov is NCT06258824.

## Guarantor

All authors accept full responsibility for the work and/or the conduct of the study. Adam Frampton and Elisa Giovannetti controlled the decision to publish. Adam Frampton, Dr. Daniel Liu and Dr. Jisce Puik had access to the data.

## Data availability statement

The datasets used and/or analysed during the current study are available from the corresponding author on reasonable request.

## Provenance and peer review

Not applicable.

## Supplementary Material

SUPPLEMENTARY MATERIAL
