# Supplementary material for: MicroRNAs as Bile-based biomarkers in pancreaticobiliary cancers (MIRABILE): a cohort study
Source: Int J Surg. 2024 Jul 23;110(10):6518–27. doi: 10.1097/JS9.0000000000001888 (PMC11486953; doi:10.1097/JS9.0000000000001888)
Supplement: SUPPLEMENTARY MATERIAL [file js9-110-6518-s001.docx]

**Supplemental Digital Content (SDC) 1, Supplementary Figures**

MicroRNAs as Bile-based Biomarkers in Pancreaticobiliary Cancers (MIRABILE)


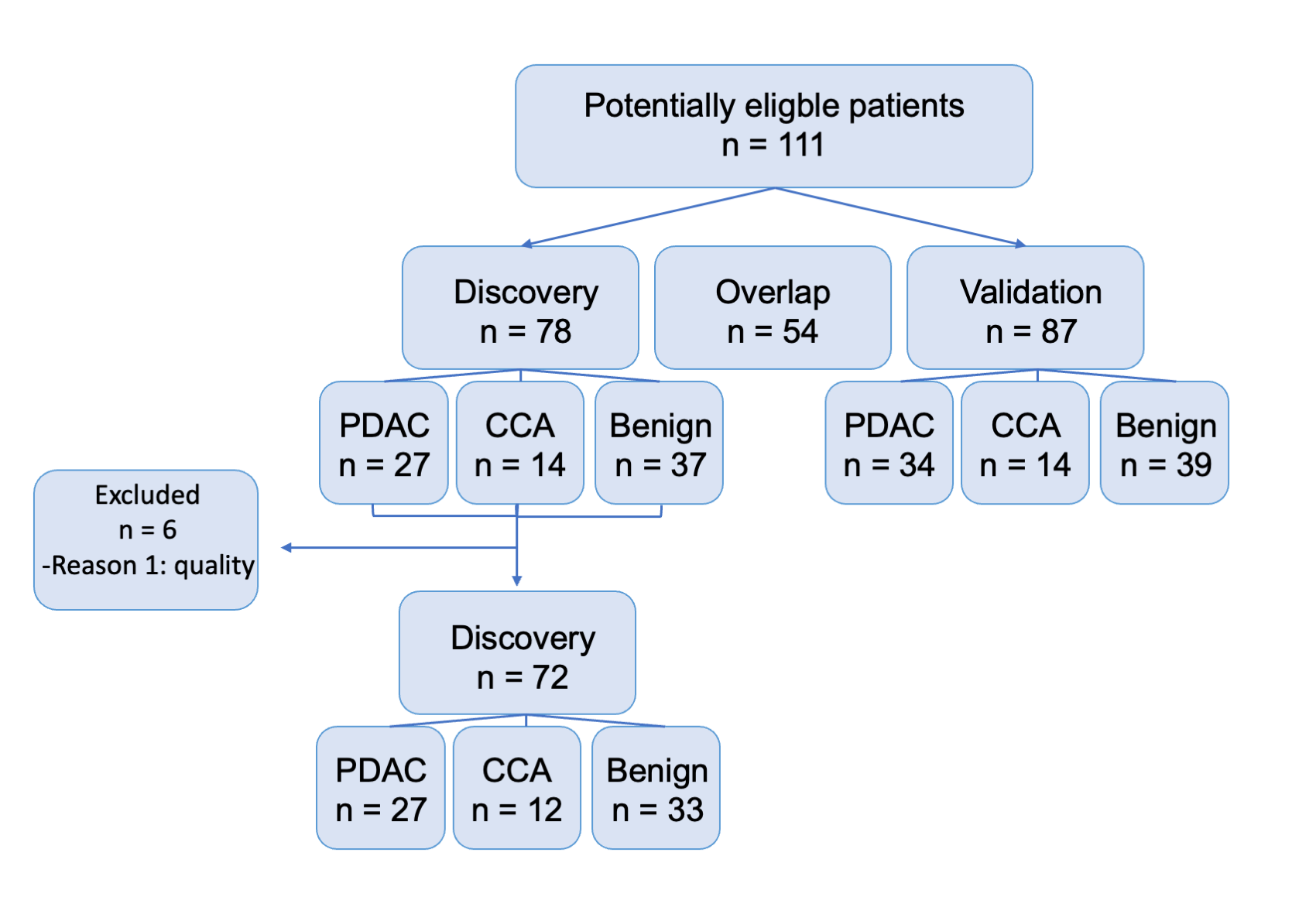


**SDC 1, Figure S1.** Flow diagram patient inclusion. CCA, cholangiocarcinoma; PDAC, pancreatic ductal adenocarcinoma.


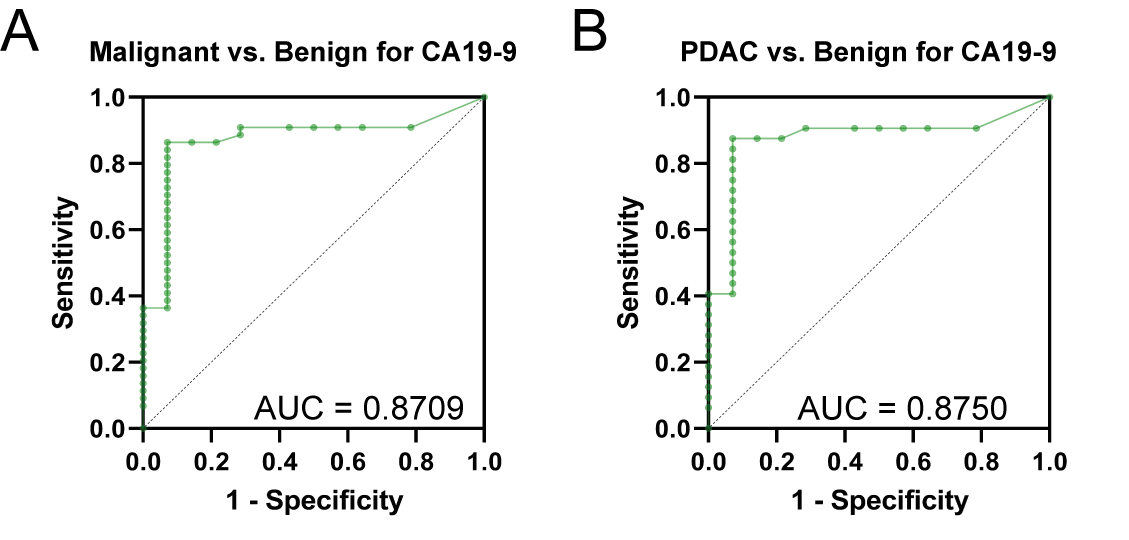


**SDC 1, Figure S2.** ROC curve analysis with corresponding AUC for diagnostic value of CA19-9 in the validation cohort. (**A**) ROC demonstrates AUC for CA19-9 for predicting malignant disease vs. benign disease. (**B**) ROC demonstrates AUC for CA19-9 for predicting PDAC vs. benign disease. AUC, area under the curve; CA 19-9, carbohydrate antigen 19-9; PDAC, pancreatic ductal adenocarcinoma; ROC, receiver operating characteristic.
