# Supplementary material for: MicroRNAs as Bile-based biomarkers in pancreaticobiliary cancers (MIRABILE): a cohort study
Source: Int J Surg. 2024 Jul 23;110(10):6518–27. doi: 10.1097/JS9.0000000000001888 (PMC11486953; doi:10.1097/JS9.0000000000001888)
Supplement: SUPPLEMENTARY MATERIAL [file js9-110-6518-s003.docx]

**Supplemental Digital Content (SDC) 3,** **Supplementary Materials and Methods**

MicroRNAs as Bile-based Biomarkers in Pancreaticobiliary Cancers (MIRABILE)

## Sample collection procedure

All patients were starved from midnight for the procedure. Bile was obtained after cannulation of the common bile duct (CBD) by ERCP and before iodine contrast injection. Using a sterile 10 ml syringe, aspiration was performed via the sphincterotome into a sterile universal container. All bile samples were transferred to the laboratory within 4 hours and flash-frozen at -80° until required. If ERCP was not technically possible, patients underwent PTC to decompress the biliary ducts. Bile was obtained from these patients within 12 hours after drainage and stored in a sterile universal container. These were also immediately transported to the lab and flash-frozen at -80°C until required.

## Clinicopathological data

Patient electronic medical records were reviewed retrospectively to obtain patient characteristics (i.e. age at time of sampling, sex), as well as information on clinical presentation and follow-up. Prior to ERCP and upon receipt of samples, concurrent serum CA 19-9, bilirubin, and CRP levels were recorded. Aetiology and tumour staging were determined in a multidisciplinary team meeting and based on standard histomorphological analysis of surgically resected specimens or biopsy specimens (open or laparoscopic surgical resection/biopsy, percutaneous needle biopsy, biliary brushing cytology, or endoscopic biopsy), and/or clinical criteria with ≥12 months of follow-up. A clinical diagnosis of malignancy was defined by the combination of a mass on radiographic imaging without acute cholangiopathy and clinical or radiographic progression after ≥12 months of follow-up, or death that was radiographically determined to be due to cancer. A benign aetiology was clinically determined on the basis of no further progression after ≥12 months follow-up with either documented resolution or stability of prior ductal abnormalities, or no further intervention as documented in electronic hospital records at 12 months.

### Preparation of samples for cell-free RNA analysis

Bile samples of 500 µL were thawed on ice, diluted with 500 µL phosphate-buffered saline (PBS) and centrifuged at 300 x *g* for 10 minutes at 4°C twice. The supernatant was transferred to a new tube while the pellet was discarded. The supernatant was then centrifuged at 16,000 x *g* for 20 minutes at 4°C twice, with the first pellet stored for later protein analysis (labelled 16k). Samples were then passed through a 0.45 µm filter (polyethersulfone) and processed immediately.

### Total RNA extraction of bile samples using TRIzol LS reagent

Total RNA from bile samples were extracted using the Invitrogen TRIzol LS (Thermo Fisher Scientific, PA, USA) according to manufacturer’s protocol (with additional chloroform and ethanol wash). To avoid contamination with RNase, filter tips and sterile Eppendorf tubes were used, and benches and work gloves were prepared for RNA extraction using RNase Zap RNase decontamination solution (Thermo Fisher Scientific). TRIzol LS Reagent was added to samples at a volumetric ratio of 3:1 and incubated for 5 minutes at room temperature to permit complete dissociation of the nucleoprotein complex. Next, 0.2 mL of chloroform per 0.75 mL of TRIzol LS Reagent was added and incubated for 5 minutes at room temperature. Separation of the mixture into a lower red phenol-chloroform, a cloudy protein interphase and a colourless upper aqueous phase was assisted by centrifugation at 12 000 x *g* at 4°C for 15 minutes. The upper aqueous phase containing RNA was transferred to a separate Eppendorf tube and 0.2 mL of chloroform per 0.75 mL of TRIzol LS Reagent was added again with incubation for 5 minutes at room temperature. The two colourless phases were separated by centrifugation at 12 000 x *g* at 4°C for 15 minutes and the upper aqueous phase was transferred to a separate Eppendorf tube.

To precipitate the RNA, 0.5 mL of isopropanol per 0.75 mL of TRIzol LS Reagent was added to the aqueous phase, incubated at RT for 5 minutes and stored at -80°C overnight (>8 hours). Samples were thawed at room temperature for 10 minutes and centrifuged at 14,000 x *g* for 30 minutes at 4°C. Total RNA precipitates formed a white pellet at the bottom of the tube and the supernatant was carefully discarded with a micropipette. The pellet was washed with 0.25 mL of 80% ethanol per 0.75 mL of TRIzol LS Reagent, vortexed and centrifuged at 7500 x *g* for 5 minutes at 4°C. This wash was repeated with 0.25 mL of 80% ethanol per 0.75 mL of TRIzol LS Reagent, vortexed and centrifuged at 7500 x *g* for 5 minutes at 4°C. Eppendorfs with RNA pellets were dried at room temperature for 20 minutes and resuspended in 15 µL RNase-free water. The solution was then transferred to a new Eppendorf tube, labelled and stored at -80°C for future analysis.

### Library preparation and next generation sequencing

RNA samples were prepared for small RNA sequencing using QIAseq small RNA Library Prep kit (Qiagen, Germany) according to manufacturer protocol. A volume of 5 µL per RNA eluate was used in low input samples (i.e. extracellular vesicles-RNA). The finished libraries were quality controlled using an Agilent Bioanalyzer 2100 (Agilent, CA, USA) and quantified by qPCR. Libraries were pooled and sequenced on an NextSeq 500 system (Illumina, San Diego, USA) using the default single-end 75 base pair protocol to include integrated unique molecular indices (UMIs).

The raw data was processed by fastx_toolkit, and Illumina adaptor sequences were removed using cutadapt^1^. Unmapped reads were mapped to miRNAs from miRBase v22 allowing zero mismatches but allowing for non-templated 3’ A and T bases. Quality control was performed using FastQC (version 0.12.0, Babraham Bioinformatics) to ensure high quality data. The miRNA read counts were subjected to differential expression analysis and normalisation using DESeq2 in R^2^. Volcano plots and other data visualisation for the RNA-Seq data was undertaken using R (Version 5 January 2015). tRNA degradation/cleavage products (due to size restriction within the library preparation step) were also analysed by differential expression analysis and normalisation using DESeq2 in R. All detected tRNA fragments were compared to tRNAs in MINTbase v2.0 (Mitochondrial and Nuclear tRNA fragment Database, an online repository of tRNA fragments found in human tissues).

### RNA quantification by RNA-specific fluorometry

Quantification of total RNA was undertaken using Qubit 2.0 Fluorometer according to manufacturer’s instructions. In short, working reagent containing an RNA-specific dye was made by diluting the Qubit RNA broad range reagent in buffer. 500 µL polypropylene tubes (Qubit assay tubes) were prepared with 190 µL of working reagent and 10 µL of standards, containing either 100 ng/µL of RNA or no RNA. 2 µL of sample was pipetted into 198 µL of working reagent and measured using the fluorometer. Concentrations were given between 0.25 to 1000 ng/µL. Although the reported lower limit of the BR assay was 1 ng/µL, all expressed values have been reported.

### RNA quality and quantity analysis using electrophoresis

RNA was quantified and (where appropriate) quality checked using an RNA 6000 Pico assay (sensitivity 50 – 5000 pg/µL) and the Agilent Bioanalyzer 2100 instrument according to manufacturer instructions. In brief, 9 µL of pre-prepared gel-dye aliquot (1 part RNA 6000 Pico dye concentrate to 65 parts filtered gel) were vortexed and spun down at 13 000 x *g* for 10 minutes at room temperature and loaded into the appropriate well of an RNA 6000 Pico chip. The chip was primed, and solutions added to appropriate wells (9 µL conditioning mix, 5 µL of marker, 1 µL of sample and 1 µL of RNA ladder) before vortexing for 60 seconds. Samples were denatured at 70°C for 2 minutes prior to measurement. Electrophoresis and measurement of total RNA was controlled using the 2100 Expert Software using ‘Eukaryotic Total RNA Pico Series II’ settings.

### Reverse Transcription Quantitative Polymerase Chain Reaction (RT-qPCR) using target-specific stem–loop primer assays

RNA was reverse transcribed using the TaqMan MicroRNA Reverse Transcription (RT) kit (Thermo Fisher Scientific, formerly Applied Biosystems). Total RNA (5 ng) made up to 2.5 μL was mixed with 3.5 μL of RT master mix (according to manufacturer instructions) and 1.5 μL of microRNA-specific 5X RT TaqMan stem–loop primers (Thermo Fisher Scientific) in a 48-well PCR plate. Samples were incubated in a Tetrad 2 96 Well Thermal Cycler (MJ Research, Deltona, USA) at 16°C for 30 minutes to allow primer annealing, followed by 30 minutes at 42°C for the elongation step and 5 minutes at 85°C to inactivate the reverse transcriptase enzyme. Specific miRNA TaqMan probes (Thermo Fisher Scientific) were used in the reverse transcription to generate specific complementary DNA (cDNA) for each sample.

Quantitative PCR reactions were performed in duplicate using miRNA specific TaqMan probes and multiple endogenous controls following manufacturer’s instructions (Thermo Fisher Scientific). MiRNA-specific cDNA samples (4 μL) was added to 10 μL of TaqMan Universal PCR master mix (2X, no uracil-N-glycosylase), 1 μL of TaqMan Assay (20X) miRNA probe assay and 5 μL of RNase-free water on a MicroAmp Fast Optical 96-well reaction plate (Thermo Fisher Scientific). This was sealed using a MicroAmp Optical Adhesive Film and run through TaqMan specific PCR cycling conditions on a StepOne Plus machine (Applied Biosystems). This involved an initial step to denature the cDNA: 50°C for 2 minutes, 95°C for 10 minutes, followed by 40 cycles of (1) denaturation at 95°C for 15 seconds and (2) annealing/elongation at 60°C for 60 seconds. Measurement of fluorescence was undertaken after each cycle and the threshold cycle (C_T_) determined by the built-in software. C_T_ values that were discordant (> 0.5 apart) were repeated as a quintuplet and the average used for further analysis. MiRNAs with a C_T_ value > 40 were deemed not detected and set at 40 for calculation of relative expression. All plates included no-template controls with any plate with a false positive repeated to ensure accuracy.

### Calculation of relative expression

Fold expression levels of candidate miRNAs were calculated using the comparative (∆∆C_T_) method and relative to the geometric mean of reference genes determined for each experiment (EndoMean). EndoMean was the geometric mean (the nth root of the product of n numbers) of values obtained for miRNAs determined as stably expressed. Relative expression levels of each miRNA were calculated using 2^-∆CT^ (∆C_T_ = C_T_ target – C_T_ EndoMean). These values were further binary logarithmically transformed and shown as Log2(fold change) expression.

**Statistical Analysis**

Statistical analyses, including descriptive statistics, were performed using GraphPad Prism v9.1.2 (GraphPad Software, CA, USA). Parametric and non-parametric clinicopathological data were analysed using the Student t-test or Mann–Whitney U test, respectively. For pairwise comparisons, clinical data that consisted of categorical variables was analysed using Fisher’s exact test. A *p* < 0.05 was considered statistically significant for pairwise comparisons of clinicopathological data.

**References**

1. Martin M. Cutadapt Removes Adapter Sequences From High-Throughput Sequencing Reads. *EMBnet journal*. 2011;17(1):10-12.

2. Love MI, Huber W, Anders S. Moderated estimation of fold change and dispersion for RNA-seq data with DESeq2. *Genome Biol*. 2014;15(12):550. doi:10.1186/s13059-014-0550-8
