# Supplementary material for: MicroRNAs as Bile-based biomarkers in pancreaticobiliary cancers (MIRABILE): a cohort study
Source: Int J Surg. 2024 Jul 23;110(10):6518–27. doi: 10.1097/JS9.0000000000001888 (PMC11486953; doi:10.1097/JS9.0000000000001888)
Supplement: SUPPLEMENTARY MATERIAL [file js9-110-6518-s004.pdf]

Supplemental Digital Content (SDC) 4, Supplementary Results

MicroRNAs as Bile-based Biomarkers in Pancreaticobiliary Cancers (MIRABILE)

Patient characteristics

Malignant and benign comparisons of demographic data showed a statistically significant difference in mean age (*p* =0.0029) in the validation cohort, corresponding with lower age at clinical presentation for benign disease. Most patients with cancer presented with large / advanced tumours (i.e. T4).Pre-therapeutic mean serum CA 19-9 levels showed no statistically significant difference when comparing malignant vs. benign disease in the discovery cohort (*p* = 0.1023), or the validation cohort (*p* = 0.0823). However, CA 19-9 values were lacking in a substantial number of patients, because patients were referred to our hospital from multiple centres, and blood tests are not routinely repeated after referral (especially not during the pandemic). Moreover, CA 19-9 measurements were not routinely conducted for patients with cholelithiasis. In total, serum CA 19-9 values were missing for 37 out of 54 patients with benign disease, and 6 out of 57 patients with malignant disease.

Furthermore, bilirubin levels were significantly higher (*p* <0.0001) in the malignant samples of both the discovery and validation cohorts. The most common cause for biliary obstruction was PDAC anatomically located in the head of pancreas (82%). Out of a total of 38 patients with PDAC, one patient had a prior stent placed and 5 had bile obtained from PTC (used in both discovery and validation, totalling 15%). In the discovery cohort, 4 out of 14 patients (28%) with CCA had a prior stent *in situ*, while in the validation cohort this was 5 out of 13 patients (38%). For the benign patients, 5 out of 37 patients (14%) had a prior stent *in situ* in the discovery cohort, while this was 17 out of 39 patients (44%) in the validation cohort.

Small RNA sequencing of bile cfRNA reveals dysregulated miRNAs in pancreaticobiliary disease

The composition of small non-coding RNAs that were present in bile samples of the discovery cohort is shown in **Fig. 2A**. MiRNAs were a highly expressed species of RNA in human bile (mean proportion of reads was 36%, range 0-85%). After quality filtering and trimming, the average total number of reads measured from bile was 15,657,465. Due to low quality, 6 samples (4 benign and 2 CCA) were omitted from further analysis, reducing the number to 72 samples. Sequencing data was filtered by miRNAs that had more than 5 sequencing reads in 8 or more samples, because not all miRNAs were uniformly expressed. This resulted in 457 miRNAs for differential expression analysis. Correlation of different samples according to miRNA expression values is shown in a principal component analysis (PCA) plot (**main text, Fig. 2B**). CCA and PDAC samples show a broad clustering of the samples with occasional outliers. In contrast, widely dispersed expression of benign samples appears to indicate heterogeneity of expression consistent with a control population. For miRNAs with adjusted *p*-values <0.01, a heatmap was generated with unsupervised hierarchical clustering according to similar expression in samples (**main text, Fig. 2C**). Receiver operating characteristic (ROC) curves with corresponding area under the curve (AUC) values were generated for the top eight miRNAs upregulated in malignant disease vs.

benign disease (miR-196a-5p, miR-184, miR-615-3p, miR-4732-3p, miR-486-5p, miR-935 and miR-1247; **SDC 4, Figure S1A**), and in PDAC vs. benign disease (miR-196-5p, miR-4732-3p, miR-615-3p, miR-935, miR-486-5p, miR-1874, miR-4732-5p,miR-215-5p; **SDC 4, Figure S1B**).

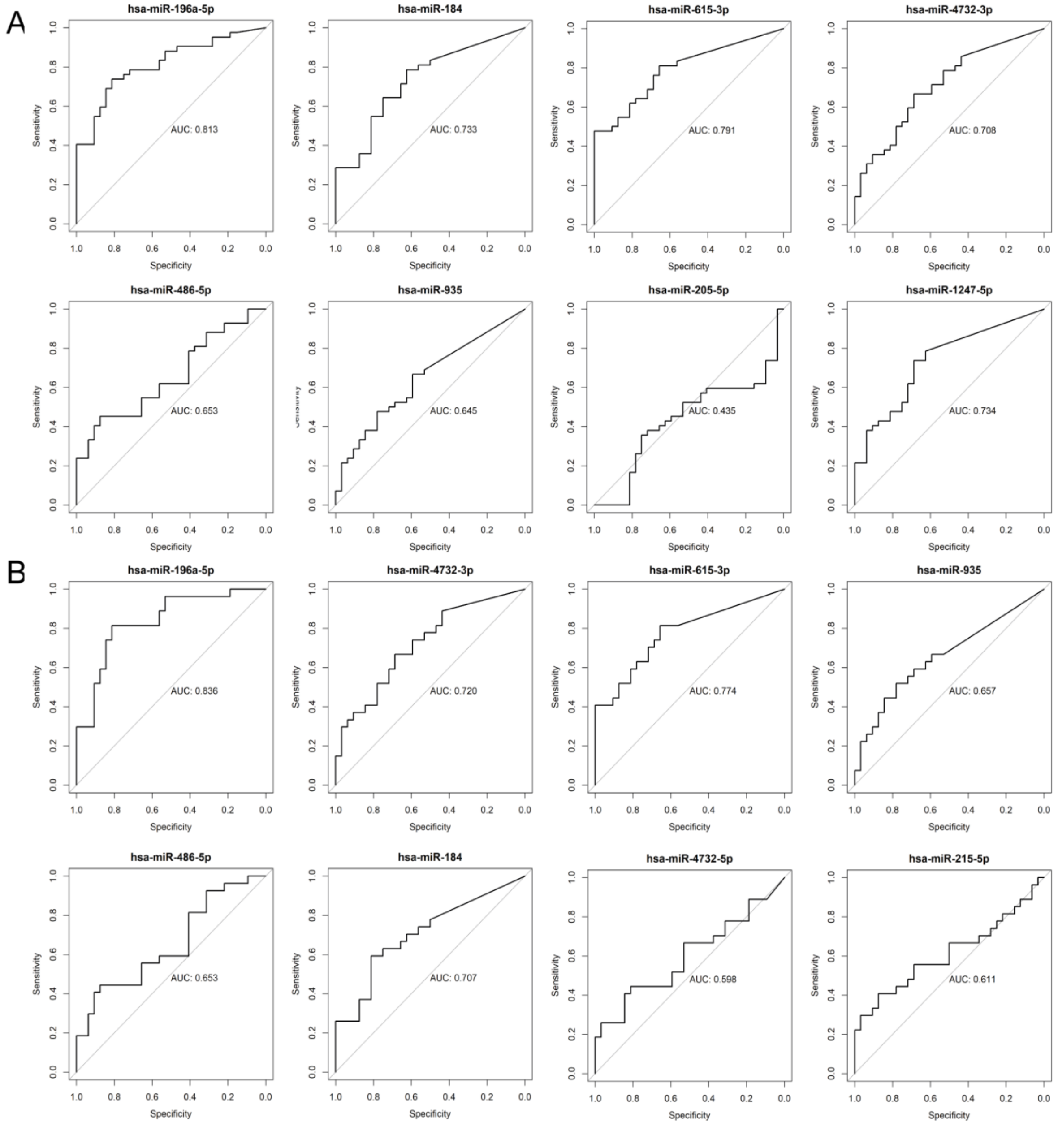

SDC 4, Figure S1. Diagnostic performance of individual miRNAs from small RNA sequencing. (**A**) Diagnostic performance displayed as ROC curves with corresponding AUC for comparing malignant and benign disease. Upper (*left to right)*: miR-196-5p, miR-184, miR-615-3p, miR-4732-3p. Below (*left to* right): miR-486-5p, miR-935, miR-205-5p and miR-4732-5p and. (**B**) Diagnostic performance displayed as ROC curves with corresponding AUC for comparing PDAC and benign disease. Upper (*left to right)*: miR-196-5p, miR-4732-3p miR-615-3p, miR-935. Below (*left to* right): miR-486-5p, miR-184, miR-4732-5p and miR-215-5p. AUC, area under the curve; CCA, cholangiocarcinoma; hsa, homo sapiens; PDAC, pancreatic ductal adenocarcinoma.

To discover miRNAs that may be associated with particular clinicopathological variables, differential miRNA expression was also assessed based on tumour location (distal vs. perihilar CCA; **SDC 4, Figure S2A**), prior stent *in situ* at ERCP (i.e. those with vs. without prior stent; **SDC 4, Figure S2B**), and resectability of the tumour in PDAC patients (**SDC 4, Figure S2C**). One miR (miR-205-5p) showed a significant upregulation in patients with no prior stents vs. prior stents *in situ* (**SDC 4, Figure S2B**). No statistically significantly dysregulated miRs were found for the other pairwise comparisons.

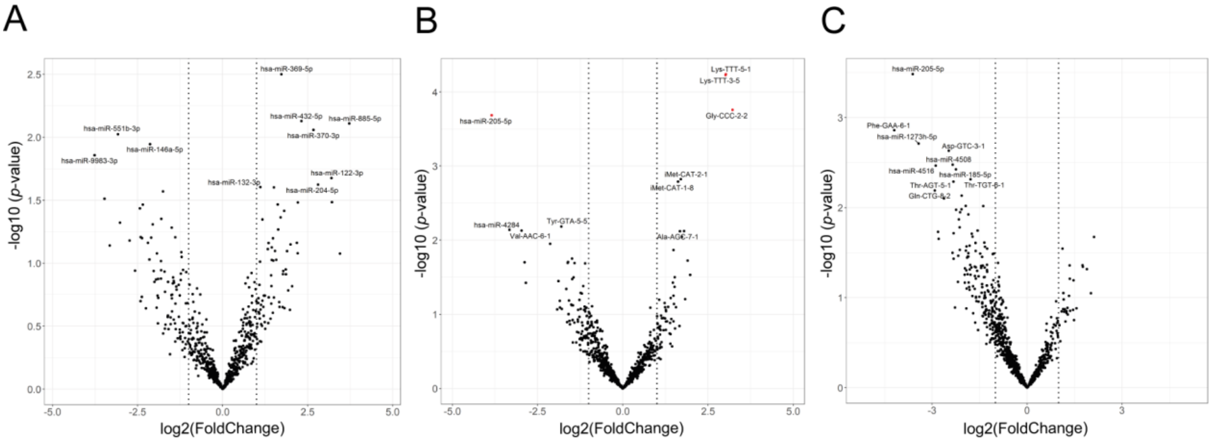

SDC 4, Figure S2. Additional volcano plots with top 10 most significant miRNAs annotated. Volcano plots are illustrated for the following pairwise comparisons (*left to right)*: (**A**) distal CCA vs. perihilar CCA – miRNA only comparison; (**B**) stent *in situ* at the time of ERCP vs. no prior stent – miRNA and tRNA comparison; (**C**) PDAC considered resectable vs. PDAC not resectable – miRNA and tRNA comparison. Red illustrates miRNAs with FDR < 0.05. Vertical dotted lines indicate log2(fold change) = +/- 1. CCA, cholangiocarcinoma; hsa, homo sapiens; PDAC, pancreatic ductal adenocarcinoma.

Small RNA sequencing also identified tRNA-derived fragments with differential expression

Although analysis of transfer RNA fragment (tRF) expression was not the aim of this study, small RNA sequencing revealed that tRFs were highly expressed in our bile cfRNA samples and on average made up 37% (range 4-84%) of the small RNA composition in bile. tRFs are derived from well-conserved 76 nucleotides (nts) long tRNAs which are cleaved into either halves (30-35 nts) or smaller fragments. These smaller fragments are defined by their relation to the original tRNA, mapping either to the 5' or 3' end, with those remaining termed 'internal tRFs'. In comparison to miRNAs, there is a higher number of experimentally validated tRFs found present in the human body (26,531 according to MINTbase v2.0). Indeed, 3176 unique tRFs with adequate expression (expressed in >50% of samples) were identified in the bile samples. While tRFs investigation was outside the scope of this study, we reckoned that preliminary results might instigate future investigations. Therefore, we performed differential expression analysis using the available sequencing data. A PCA plot and corresponding heatmap of differentially expressed tRFs are shown in **SDC 4, Figure S3**.

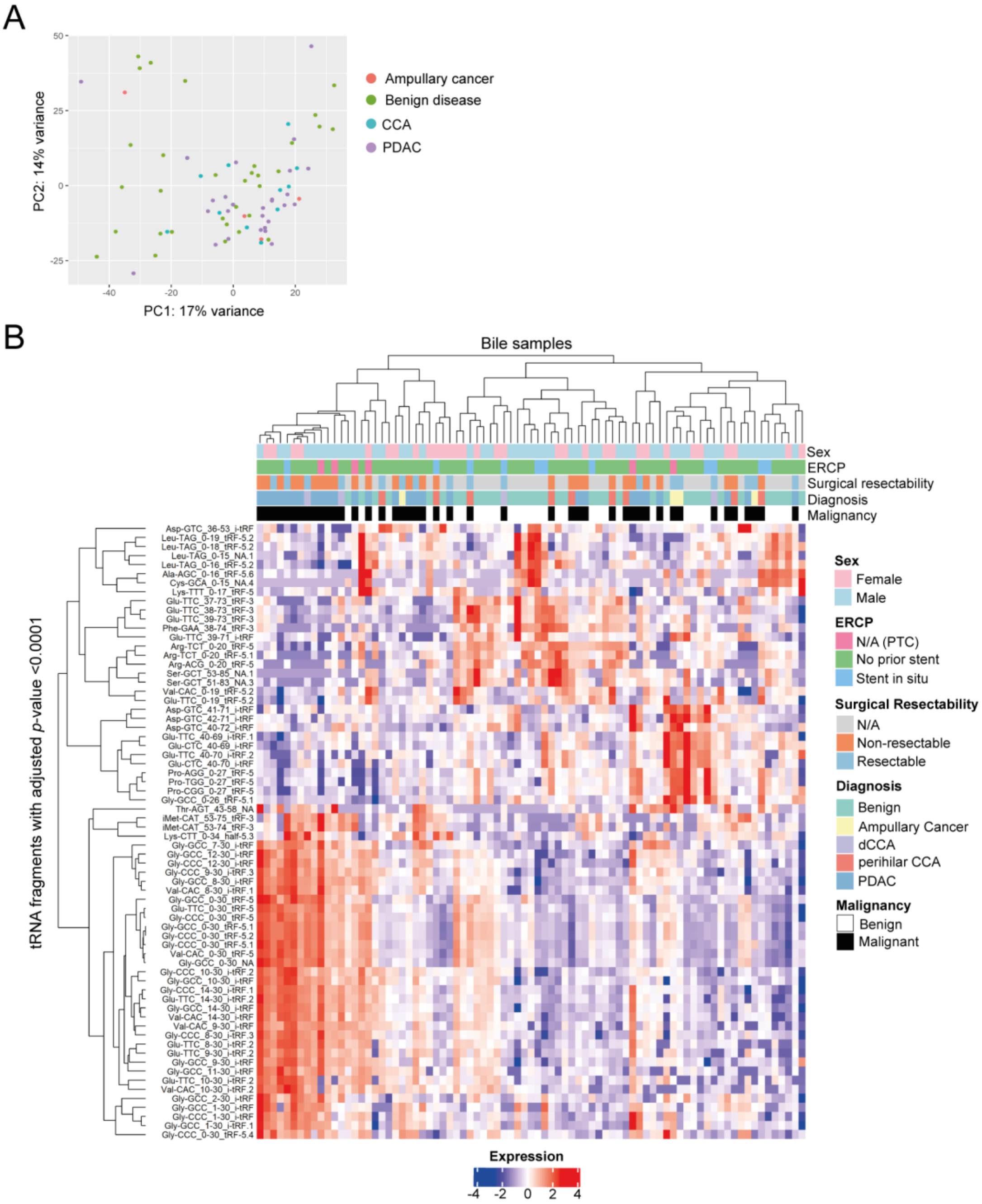

SDC 4, Figure S3. Differential expression analysis identified significant tRFs present in bile samples. (**A**) Principal component analysis plot of bile tRF expression showing grouping of the samples. (**B**) Heatmap with unsupervised hierarchical clustering showing all tRFs with adjusted *p*-value <0.0001. CCA, cholangiocarcinoma; mRNA, messenger RNA; N/A, not applicable; PC, principal component; PDAC, pancreatic ductal adenocarcinoma; PTC, percutaneous transhepatic cholangiography; rRNA, ribosomal RNA; snRNA, small nuclear RNA; tRNA, transfer RNA.

When comparing malignant and benign bile samples, 264 differentially expressed tRFs were identified, of which 165 were upregulated in malignant disease (i.e. miRNAs deemed detectable in

cancer and possibly of higher interest as they may be easier to use as diagnostic biomarker; **SDC 4, Figure S4A**). The top 10 tRFs that were upregulated in malgingant vs. benign disease are displayed in **SDC 4, Table S4**. Gly-GCC_0-30_trF-5 demonstrated a high statistical significance (adjusted *p*-value = 6.04E-09) and fold change (3.3), as shown in the volcano plot for the pairwise comparisons of malignant vs. benign (**SDC 4, Figure S4B)**. Furthermore, 474 tRFs were differentially expressed in PDAC compared to benign disease, 65 in CCA versus benign disease, and 124 in PDAC vs. CCA (**SDC 4, Figure S4C**).

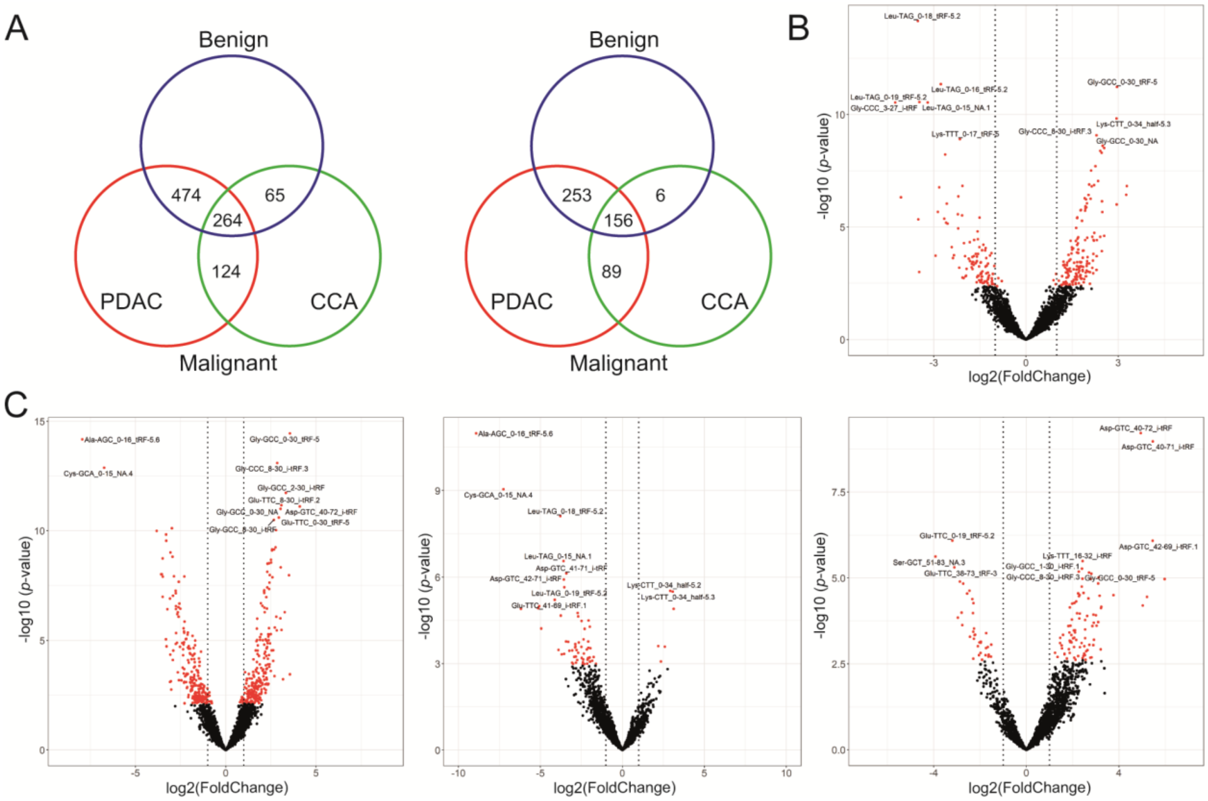

SDC 4, Figure S4. tRFs with statistically significant differential expression in bile samples. (**A**) Venn diagram showing the total number of differentially expressed tRFs (*left*) and total number of upregulated tRFs only (*right*) between PDAC, CCA and benign cohorts with an adjusted p-value <0.05 in bile. Volcano plots with top 10 statistically most significant tRFs annotated for the pairwise comparisons: (**B**) malignant vs. benign disease; (**C**, *left*) PDAC vs benign disease; (**C**, *middle*) CCA vs benign disease; (**C**, *right*) PDAC vs CCA. Red illustrates miRNAs with FDR < 0.05. Vertical dotted lines indicate log2(fold change) = +/- 1. CCA, cholangiocarcinoma; hsa, homo sapiens; PDAC, pancreatic ductal adenocarcinoma.

SDC 4, Table S4. Top ten transfer RNA fragments identified as differentially expressed in malignant compared to benign bile samples

tRNA fragment	MINTbase Unique ID	Expression	Log2(FoldChange)	Standard Error	Adjusted *p*-value
Gly-GCC_52-71_i-tRF	tRF-19-WE8SPOJU	6.4752	3.2842	0.6253	2.18 E-05
Gly-CCC_37-70_i-tRF	tRF-33-WYL1M3WE8S68DM	4.7185	3.2624	0.6417	4.56 E-05
Gly-GCC_0-30_tRF-5	tRF-30-P4R8YP9LON4V	124300.0	2.9616	0.4304	6.04 E-09
Thr-AGT_43-58_NA	NA	5.0630	2.9532	0.6036	8.94 E-05
Lys-CTT_0-34_half-5.3	tRF-34-PSQP4PW3FJIKE5	112.2100	2.9451	0.4599	6.61 E-08
Glu-TTC_8-30_i-tRF.2	tRF-22-R918VBY9M	11.1660	2.5461	0.4290	8.49 E-07
Asp-GTC_28-50_i-tRF	tRF-22-L7S5QKF14	6.0747	2.5421	0.6024	0.001308
Gly-GCC_0-30_NA	NA	548.8500	2.4962	0.4186	7.55 E-08
Gly-TCC_37-52_NA	NA	2.8600	2.4858	0.5249	0.000158
Val-CAC_0-34_half-5	tRF-34-Q99P9P9NH57S15	113.1500	2.4714	0.4987	7.09 E-05

This table shows the top ten tRNA fragment candidates identified by RNA sequencing in malignant vs benign, sorted by ascending log fold change values. Although tRF functionalities are yet to be fully uncovered, evidence has already been provided for their oncogenic and tumour suppressor roles in several cancers[1]. In this study, after multiple hypothesis correction, 264 tRFs (out of the initial 3176 significant tRFs) were identified differentially expressed between malignant and benign disease, including Gly-GCC_0-30_trF-5. Further differential analysis identified 474 significant tRFs comparing PDAC and benign disease, and 65 comparing CCA and benign disease. Several significant tRFs differed only by one or a short number of nucleotides and yet showed upregulated expression levels individually. Gly-GCC and Glu-CTC were the most dominant isotypes in bile. Possibly, these isotopes share a functional target with overlapping sequence homology. Abbreviations: i-tRF, internal tRF; NA, unassigned tRF; tRNA, transfer RNA.

Although some fragments varied only by a short number of nucleotides, they could show upregulated expression levels individually. For example, Gly-GCC_0-30_tRF-5 and Gly-GCC_0-30_NA varied by a single nucleotide and were both upregulated in malignant disease (**SDC 4, Table S4**). Similarly, Asp-GTC_40-71_i-tRF and Asp-GTC_40-72_i-tRF vary by one nucleotide and showed upregulation in PDAC. The most dominant tRNA isotypes (i.e. the tRNA from which the fragment is likely to have arisen) in bile were Gly-GCC and Glu-CTC (**SDC 4, Figure S5**). It may be that these molecules share a functional target with overlapping sequence homology which is yet to be elucidated.

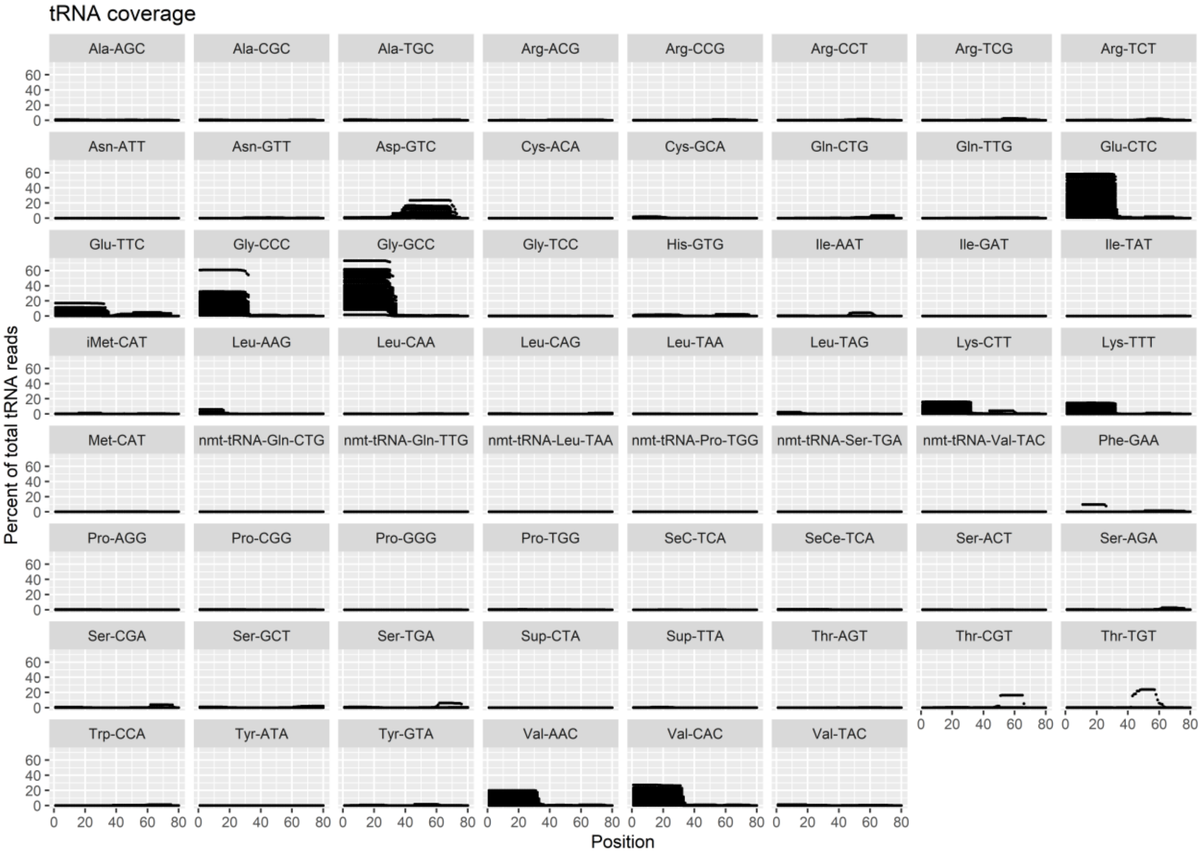

SDC 4, Figure S5. Normalised read distribution of tRFs in bile according to tRNA isotypes (tRNA of origin). Expression as a percentage of total tRNA reads with relative nucleotide position within the tRNA expressed along the y-axis. tRNA, transfer RNA.

Generating a diagnostic miRNAs signature using LASSO regression analysis

For an unbiased approach in the selection of candidate bile miRNAs from sequencing data, all miRNAs with adjusted $p < 0.05$ were used as input variables for LASSO regression analysis. To improve the model, candidate miRNAs were filtered by expression level, requiring candidates to be detected with 10,000 or more normalized reads across all bile samples. This final model led to a combination of 14 bile miRNAs that could discriminate malignant from benign disease with an AUC of 0.93 (95%CI 0.84-1.00; **SDC 4, Figure S6A**). Furthermore, a 7-miRNA combination (miR-196a-5p, miR-199a-5p, miR-335-5p, miR-182-5p, miR-146a-5p, miR-345-5p and miR-222-3p) showed an AUC of 1.00 (95%CI 1.00-1.00) in distinguishing PDAC from benign disease (**SDC 4, Figure S6B**). Corresponding heatmaps are included in **SDC 4, Figure S7**. Six upregulated miRNAs (i.e. those that were considered upregulated and expressed in cancer may be easier to apply as cancer biomarker) were selected for RT-qPCR validation: miR-196a-5p, miR-199a-5p, miR-335-5p, miR-182-5p, miR-340-5p and miR-424-5p. These six miRNAs were selected because a) they were all included in the 14-miRNA model to differentiate between malignant and benign disease, and b) the first four miRNAs were also included in the LASSO regression model to discriminate PDAC from benign disease.

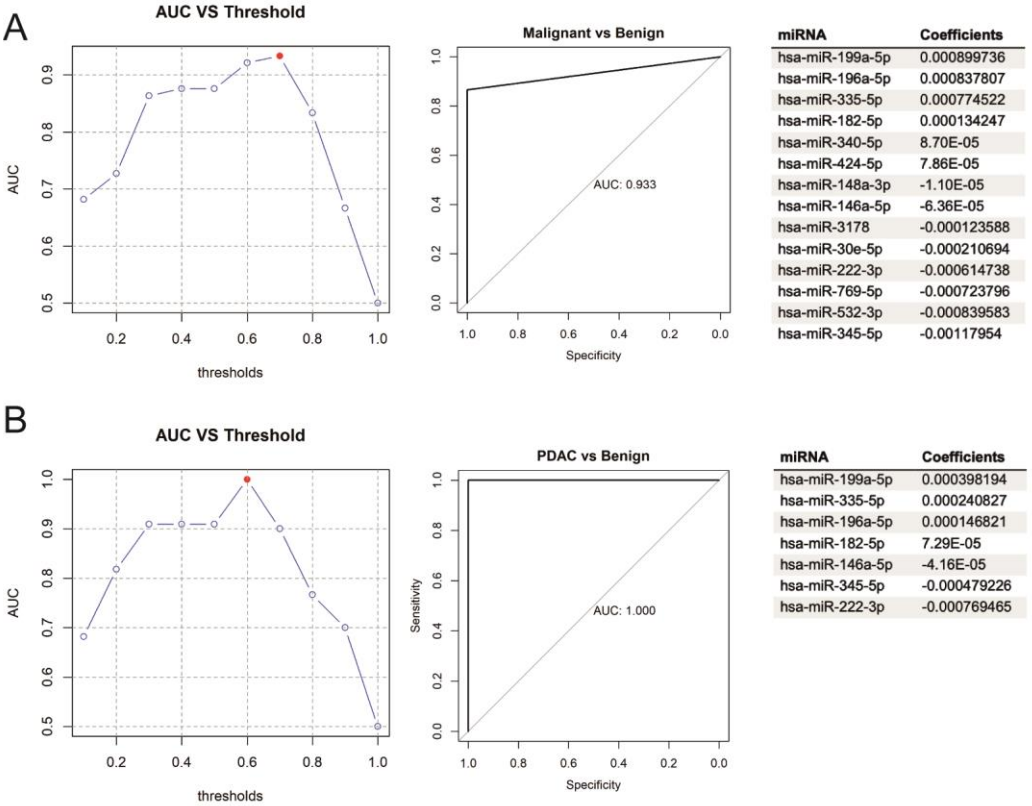

SDC 4, Figure S6. LASSO miRNA signatures for prediction of malignant disease. (**A**) Graph plot demonstrating different thresholds and their effect on the AUC (*left*). ROC curves were generated using the predetermined thresholds at maximum Youden Index for the pairwise comparison of malignant versus benign disease, with potential AUC = 0.93 (*middle*) for miRNAs shown in the adjacent table (*right*). (**B**) Graph plot demonstrating different thresholds and their effect on the AUC for PDAC versus benign disease (*left*), with potential AUC = 1.00

(*middle*) for miRNAs shown in the adjacent table (*right*). AUC, area under the curve; CCA, cholangiocarcinoma; hsa, homo sapiens; PDAC, pancreatic ductal adenocarcinoma.

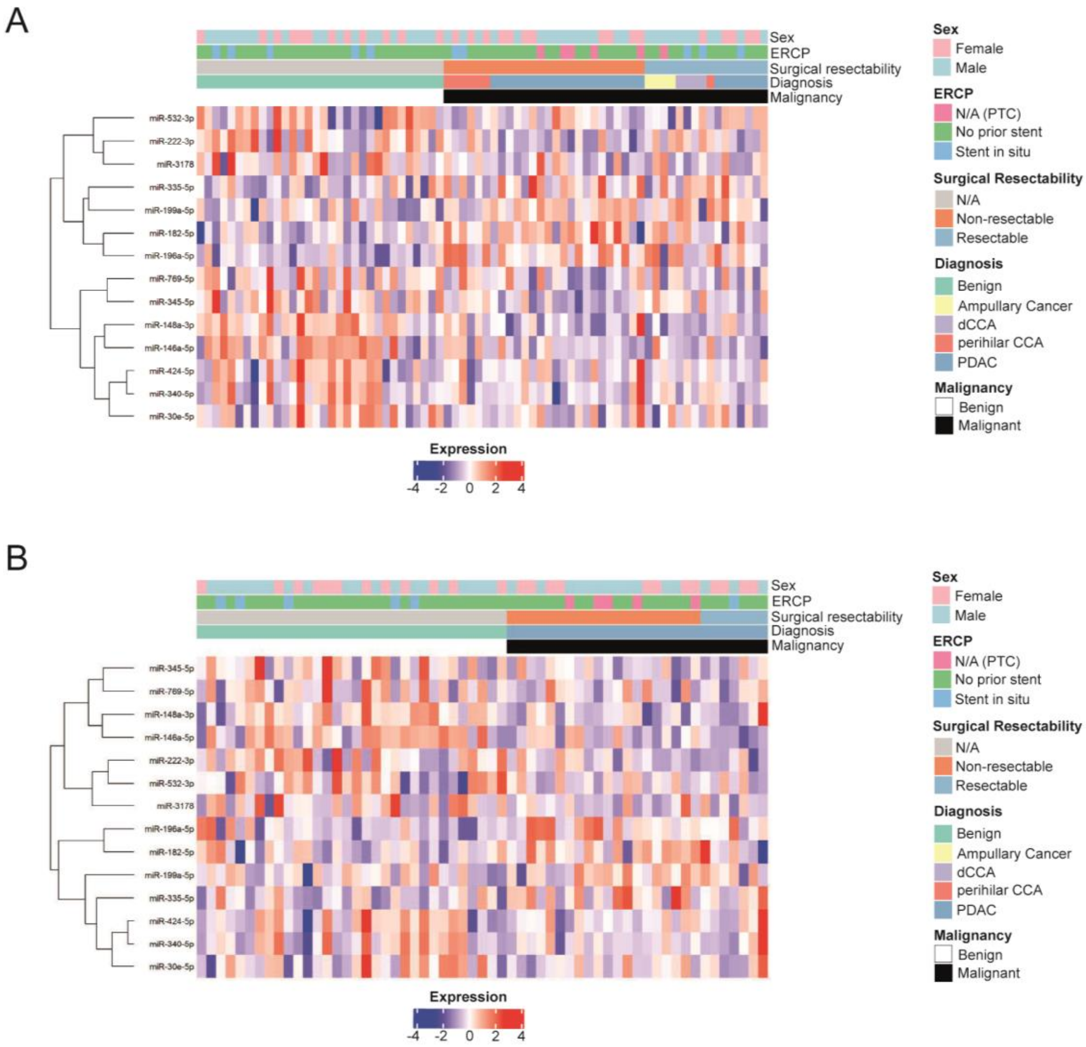

SDC 4, Figure S7. Heatmaps for candidate miRNA signatures generated by LASSO regression analysis. (**A**) Expression of LASSO signature miRNAs in all bile samples. (**B**) Expression of LASSO signature miRNAs in PDAC and benign bile samples. CCA, cholangiocarcinoma; dCCA, distal CCA; ERCP, Endoscopic retrograde cholangiopancreatography; N/A, not applicable; PDAC, pancreatic ductal adenocarcinoma; PTC, percutaneous transhepatic cholangiography.

Correlation analysis of candidate miRNAs and laboratory markers

The selection of 4 candidate miRNAs (i.e. those that were considered upregulated and expressed in cancer may be more pragmatic to apply as cancer biomarker) were analysed by RT-qPCR. Since strong collinearity between input variables (i.e. miRs) for logistic regression influences the accurate estimation of the miRNA model, Spearman's Rank correlation analysis was performed for miRs and several laboratory markers, including bilirubin, CRP and CA 19-9 (**SDC 4, Figure S8**). The 4 miRNAs showed low correlation with each other (between -0.26 and 0.50), indicating that they could be used in a final model. Bilirubin was positively correlated with CA 19-9 (rs:0.51; *p* <0.001).

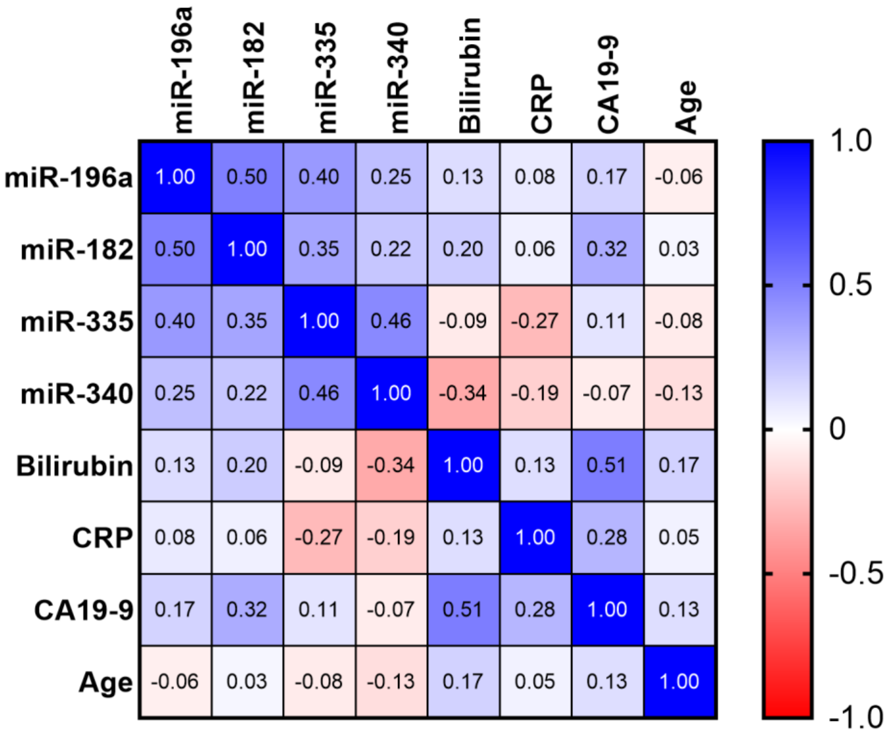

SDC 4, Figure S8. Correlation analysis of miRNAs of interest, laboratory markers and age. Heatmap showing Spearman's rank correlation coefficients, where 1.0 is perfect positive correlation and -1.0 is perfect negative correlation. CA 19-9, carbohydrate antigen 19-9; CRP, C-reactive protein.

Functional Analysis of candidate miRNAs to identify common pathways

MiEAA, an online web-based tool that utilizes the GeneTrail toolkit and the database miRwalk2.0, was used to perform Gene Set Enrichment Analysis (GSEA) of the six candidate miRNAs (including those that could not be validated by RT-qPCR)[2]. This is a computational approach used to determine whether known biological functions or processes are statistically over-represented within the experimentally derived list[3]. *P*-values were calculated and adjusted for multiple comparisons. We identified 40 functional pathways for all 6 candidates with an adjusted *p*-value <0.05. The top ten is shown in **SDC 5, Table S5**. This analysis highlighted pathways including bone morphogenetic proteins (BMP) signalling, Toll-like receptor (TLR) pathways and involvement of the endosome. BMPs regulate developmental epithelial-to-mesenchymal Transition (EMT) and there is evidence that there is a role for BMP signalling in promoting the metastatic cascade[4].

References

1. Di Fazio A, Gullerova M. An old friend with a new face: tRNA-derived small RNAs with big regulatory potential in cancer biology. *British Journal of Cancer*. 2023/05/01 2023;128(9):1625-1635. doi:10.1038/s41416-023-02191-4

2. Kern F, Fehlmann T, Solomon J, et al. miEAA 2.0: integrating multi-species microRNA enrichment analysis and workflow management systems. *Nucleic Acids Res*. Jul 2 2020;48(W1):W521-w528. doi:10.1093/nar/gkaa309

3. Draghici S, Khatri P, Martins RP, Ostermeier GC, Krawetz SA. Global functional profiling of gene expression. *Genomics*. Feb 2003;81(2):98-104. doi:10.1016/s0888-7543(02)00021-6

4. Gordon KJ, Kirkbride KC, How T, Blobe GC. Bone morphogenetic proteins induce pancreatic cancer cell invasiveness through a Smad1-dependent mechanism that involves matrix metalloproteinase-2. *Carcinogenesis*. Feb 2009;30(2):238-48. doi:10.1093/carcin/bgn274
