# Supplementary material for: MicroRNAs as Bile-based biomarkers in pancreaticobiliary cancers (MIRABILE): a cohort study
Source: Int J Surg. 2024 Jul 23;110(10):6518–27. doi: 10.1097/JS9.0000000000001888 (PMC11486953; doi:10.1097/JS9.0000000000001888)
Supplement: SUPPLEMENTARY MATERIAL [file js9-110-6518-s005.docx]

**Supplemental Digital Content (SDC) 5, Supplementary Tables**

MicroRNAs as Bile-based Biomarkers in Pancreaticobiliary Cancers (MIRABILE)

| **SDC 5, Table S1.** Top twenty microRNAs identified as differentially expressed in malignant (pancreatic ductal adenocarcinoma and cholangiocarcinoma) bile samples | | | | | | | | | |
| --- | --- | --- | --- | --- | --- | --- | --- | --- | --- |
| **MicroRNA** | **Expression** | | **Log2(FoldChange)** | | **Standard Error** | | **Adjusted *p-*value** | | |
| hsa-miR-196a-5p | 557. | 52 | 5. | 12 | 0. | 57 | 1. | 56 | E-16 |
| hsa-miR-3178 | 493. | 50 | -4. | 28 | 0. | 65 | 9. | 25 | E-09 |
| hsa-miR-1246 | 1527. | 50 | -3. | 81 | 0. | 66 | 7. | 15 | E-07 |
| hsa-miR-184 | 10. | 16 | 3. | 27 | 0. | 60 | 3. | 18 | E-06 |
| hsa-miR-1273h-5p | 8. | 57 | -3. | 25 | 0. | 64 | 7. | 15 | E-07 |
| hsa-miR-615-3p | 5. | 47 | 3. | 11 | 0. | 51 | 3. | 18 | E-06 |
| hsa-miR-6724-5p | 23. | 38 | -3. | 05 | 0. | 50 | 1. | 08 | E-07 |
| hsa-miR-4484 | 24. | 62 | -2. | 99 | 0. | 63 | 6. | 64 | E-05 |
| hsa-miR-142-5p | 3243. | 57 | -2. | 99 | 0. | 48 | 7. | 39 | E-08 |
| hsa-miR-4732-3p | 28. | 75 | 2. | 86 | 0. | 62 | 0. | 000116 | |
| hsa-miR-486-5p | 24172. | 64 | 2. | 68 | 0. | 46 | 6. | 90 | E-07 |
| hsa-miR-935 | 6. | 71 | 2. | 67 | 0. | 67 | 0. | 001142 | |
| hsa-miR-205-5p | 140. | 20 | 2. | 53 | 0. | 52 | 4. | 63 | E-05 |
| hsa-miR-1247-5p | 7. | 80 | 2. | 48 | 0. | 61 | 0. | 000975 | |
| hsa-miR-10396b-3p | 11. | 10 | -2. | 40 | 0. | 31 | 0. | 015332 | |
| hsa-miR-206 | 6. | 82 | -2. | 34 | 0. | 56 | 0. | 000637 | |
| hsa-miR-3175 | 6. | 09 | -2. | 32 | 0. | 67 | 0. | 006251 | |
| hsa-miR-4443 | 4. | 91 | -2. | 30 | 0. | 63 | 0. | 003616 | |
| hsa-miR-4668-5p | 14. | 26 | -2. | 26 | 0. | 55 | 0. | 000855 | |
| hsa-miR-4732-5p | 57. | 66 | 2. | 25 | 0. | 51 | 0. | 000337 | |
| Top twenty miRNA candidates (adjusted *p*<0.05) identified by RNA sequencing sorted by log fold change. Expression levels were averaged across all samples with normalization by DESeq2 to allow direct comparison between the samples. Standard errors are of the log2fold change and p-values are adjusted using the Benjamini-Hochberg method. In bold are candidates used in later validation. hsa, homo sapiens. | | | | | | | | | |

| **SDC 5, Table S2.** Top ten microRNAs identified as differentially expressed in pancreatic ductal adenocarcinoma compared to benign bile samples | | | | | | | | | |
| --- | --- | --- | --- | --- | --- | --- | --- | --- | --- |
| **MicroRNA** | **Expression** | | **Log2(FoldChange)** | | **Standard Error** | | **Adjusted *p-*value** | | |
| hsa-miR-196a-5p | 557. | 52 | 4. | 55 | 0. | 63 | 3. | 48 | E-10 |
| hsa-miR-3178 | 493. | 50 | -4. | 44 | 0. | 71 | 8. | 84 | E-08 |
| hsa-miR-6724-5p | 23. | 38 | -3. | 53 | 0. | 56 | 5. | 67 | E-08 |
| hsa-miR-4732-3p | 28. | 75 | 3. | 28 | 0. | 67 | 8. | 01 | E-05 |
| hsa-miR-4484 | 24. | 62 | -3. | 23 | 0. | 71 | 0. | 00022844 | |
| hsa-miR-615-3p | 5. | 47 | 3. | 16 | 0. | 56 | 2. | 10 | E-06 |
| hsa-miR-935 | 6. | 72 | 2. | 90 | 0. | 74 | 0. | 00149038 | |
| hsa-miR-1273h-5p | 8. | 57 | -2. | 88 | 0. | 72 | 0. | 00138192 | |
| hsa-miR-486-5p | 24172. | 65 | 2. | 82 | 0. | 52 | 5. | 87 | E-06 |
| hsa-miR-10401-5p | 12. | 68 | -2. | 79 | 0. | 66 | 0. | 00076830 | |
| Top ten miRNA candidates (adjusted *p*<0.05) identified by RNA sequencing in PDAC vs benign, sorted by log fold change values. hsa, homo sapiens. | | | | | | | | | |

| **SDC 5, Table S3.** Top ten microRNAs identified as differentially expressed in cholangiocarcinoma compared to benign bile samples | | | | | | | | | |
| --- | --- | --- | --- | --- | --- | --- | --- | --- | --- |
| **MicroRNA** | **Expression** | | **Log2(FoldChange)** | | **Standard Error** | | **Adjusted *p-*value** | | |
| hsa-miR-196a-5p | 557.. | 52 | 5. | 75 | 0. | 84 | 5. | 58 | E-09 |
| hsa-miR-3178 | 493. | 50 | -5. | 54 | 0. | 95 | 2. | 05 | E-06 |
| hsa-miR-184 | 10. | 16 | 3. | 84 | 0. | 85 | 0. | 00077565 | |
| hsa-miR-1273h-5p | 8. | 57 | -3. | 77 | 0. | 98 | 0. | 00790652 | |
| hsa-miR-619-5p | 14. | 54 | -3. | 73 | 0. | 06 | 0. | 02079502 | |
| hsa-miR-137-3p | 2. | 80 | 3. | 64 | 0. | 74 | 0. | 00021606 | |
| hsa-miR-206 | 6. | 82 | -3. | 37 | 0. | 83 | 0. | 00368810 | |
| hsa-miR-205-5p | 140. | 20 | 3. | 36 | 0. | 76 | 0. | 00117353 | |
| hsa-miR-615-3p | 5. | 47 | 3. | 28 | 0. | 71 | 0. | 00068175 | |
| hsa-miR-3175 | 6. | 09 | -3. | 28 | 0. | 02 | 0. | 03893486 | |
| Top ten miRNA candidates (adjusted *p*<0.05) identified by RNA sequencing in CCA vs benign, sorted by log fold change values. hsa, homo sapiens. | | | | | | | | | |

| **SDC 5, Table S4.** Identification of individual endogenous normalisers using Normfinder algorithm | | | | | | |
| --- | --- | --- | --- | --- | --- | --- |
| **MicroRNA** | **GroupDif** | | **GroupSD** | | **Stability** | |
| miR-339-3p | 0. | 56 | 0. | 79 | 0. | 14 |
| miR-500a-3p | 0. | 88 | 0. | 62 | 0. | 16 |
| miR-30e-3p | 0. | 87 | 0. | 76 | 0. | 17 |
| miR664a-5p | 0. | 38 | 0. | 89 | 0. | 17 |
| miR345-5p | 1. | 10 | 0. | 78 | 0. | 17 |
| miR-30d-5p | 0. | 85 | 0. | 81 | 0. | 17 |
| miR-186-5p | 0. | 59 | 0. | 72 | 0. | 17 |
| miR-532-5p | 0. | 72 | 0. | 78 | 0. | 18 |
| miR-598-3p | 0. | 22 | 0. | 82 | 0. | 18 |
| miR-222-3p | 0. | 85 | 0. | 64 | 0. | 18 |
| Let-7d-5p | 0. | 47 | 0. | 85 | 0. | 20 |
| miR-362-5p | 0. | 65 | 0. | 93 | 0. | 21 |
| miR-24-3p | 0. | 53 | 0. | 58 | 0. | 21 |
| miR-28-3p | 0. | 82 | 0. | 87 | 0. | 21 |
| miR-132-3p | 0. | 51 | 0. | 82 | 0. | 21 |
| miR-148a-3p | 1. | 35 | 0. | 73 | 0. | 21 |
| miR-148b-3p | 0. | 44 | 0. | 85 | 0. | 21 |
| miR-324-5p | 0. | 69 | 0. | 93 | 0. | 21 |
| Let-7b-5p | 0. | 51 | 0. | 67 | 0. | 21 |
| miR-320a-3p | 0. | 62 | 0. | 99 | 0. | 21 |
| MiRNAs identified as stably expressed and sorted by descending stability. GroupDif is the estimated difference in expression between the groups; GroupSD is a weighted average of the estimated intra-group variation and Stability is the Normfinder expression stability measure with lower values indicating stable gene expression. | | | | | | |

| **SDC 5, Table S5.** Top ten functional pathways for all 6 candidate miRNAs | | | | | | | |
| --- | --- | --- | --- | --- | --- | --- | --- |
| **Subcategory** | | **Enrichment** | ***P-*value** | | | **Adjusted *p-*value** | |
| GO0030509 | BMP Signalling Pathway | Over-represented | 1. | 47 | E-05 | 0. | 0206292 |
| GO0007595 | Lactation | Over-represented | 6. | 94 | E-05 | 0. | 0206292 |
| GO0009791 | Post Embryonic Development | Over-represented | 8. | 89 | E-05 | 0. | 0206292 |
| GO0005768 | Endosome | Over-represented | 1. | 30 | E-04 | 0. | 0206292 |
| GO0003705 | RNA Polymerase II DNA Enhancer Sequence Specific DNA Binding Transcription Factor Activity | Over-represented | 1. | 48 | E-04 | 0. | 0206292 |
| GO0002224 | Toll Like Receptor Signalling Pathway | Over-represented | 1. | 55 | E-04 | 0. | 0206292 |
| GO0034130 | Toll Like Receptor 1 Signalling Pathway | Over-represented | 1. | 55 | E-04 | 0. | 0206292 |
| GO0034134 | Toll Like Receptor 2 Signalling Pathway | Over-represented | 1. | 55 | E-04 | 0. | 0206292 |
| GO0005635 | Nuclear Envelope | Over-represented | 1. | 69 | E-04 | 0. | 0206292 |
| GO0008063 | Toll Signalling Pathway | Over-represented | 1. | 69 | E-04 | 0. | 0206292 |
| Top ten functional pathways that were statistically over-represented in all 6 candidate miRNAs (miR-199a-5p, miR-196a-5p, miR-335-5p, miR-182-5p, miR-340-5p and miR-424-5p). | | | | | | | |

| **SDC 5, Table S6.** Literature overview of studies on bile miRNAs | | | | | |
| --- | --- | --- | --- | --- | --- |
| **First author** | **Year** | **Number of patients** | **EV study** | **Candidate Bile miRNAs** | **Comments** |
| **Shigahera et al.^1^** | 2011 | 7 CCA  2 Gallbladder cancer  9 Benign | No | miR-9, miR-302c*, miR-199a-3p, miR-222*, miR-145*, miR-105, miR-942, miR-147b, let-7f-2*, let-7i* | AUC of miR-9 was 0.975. |
| **Cote et al.^2^** | 2014 | Discovery  25 PDAC  35 Benign  Validation  28 PDAC  61 Benign | No | miR-10b, miR-30c, miR-106b, miR-155, miR-212 | Selection of candidate miRNAs based on literature. AUC for 5-miRNA panel was AUC>9.90. |
| **Li et al.^3^** | 2014 | Discovery  3 CCA  3 Benign  Validation  46 CCA  50 Benign | Yes | miRNA panel miR-191, miR-468-3p, miR-1247b, miR-16, miR-484 | Cel-miR-39 used as normaliser. 5-miRNA panel showed sensitivity of 67% and specificity of 96%. |
| **Voigtländer et al.^4^** | 2015 | Discovery  15 PSC  8 CCA  Validation  52 PSC  12 PSC/CCA  19 CCA | No | miR-412, miR-640, miR-1537, miR-3189 | Normalised to global mean in screening & spike-in C. elegans. Array-based screening. Combination with CA19-9 improved AUC to 0.91. |
| **Le et al.^5^** | 2019 | 14 CCA  12 PDC  09 Benign | No | miR-16, miR-221 and miR-196a  (brush cytology) | MiRNA selection based on literature. Combination of miR-196a together with cytology reached a sensitivity of 92% and specificity of 100% |
| **Han et al.^6^** | 2019 | Discovery  11 CCA  10 Benign  Validation  37 CCA  48 Benign | No | miR-30d-5p, miR-92a-3p | Used array data to identify candidate miRNAs. Combination with CA19-9 resulted in an AUC of 0.689. |
| **Han et al.^7^** | 2021 | 1 CCA  1 Benign | Yes | miR-10a-5p, miR-215-5p, miR-451a, miR-200b-3p, miR-181a-5p, miR-7-5p, miR-29b-3p, miR-29a-3p, let-7a-3p, miR-429, miR-21-5p, miR-192-5p, miR29c-3p, miR574-5p, miR-191-5p, miR23a-5p, miR-619-5p, miR-8485, miR-15a-3p, miR1273c, miR7847-3p, miR-1285-3p | Used next generation sequencing to identify candidate miRNAs. No ROC analysis. |
| **Ge et al.^8^** | 2021 | Discovery  5 Malignant  5 Benign  Validation  12 PDAC  15 CCA  10 Ampullary cancer  45 Benign | Yes | mir-483-5p, mir-126-3p | The combination of mir-483-5p and mir-126-3p showed an AUC of 0.81, which was higher than the AUC for CA19-9 (0.74). |
| **Uchihata^9^** | 2022 | 53 CCA  31 Benign | No | miR-31-5p, miR-378d, miR-182-5p, and miR-92a-3p  (brush cytology/ bile smear slides) | RNU6 was used as normaliser. MiRNA selection was based on literature of lung cancer. MiR-182-5p in bile cytologic samples showed an AUC of 0.86. |
| **Yoshida et al.^10^** | 2022 | Discovery  11 Biliary tract cancers  9 Benign  Validation  34 Biliary tract cancers  34 Benign | Yes | miR-451a, miR-21-5p, miR-3619-3p, miR-6778-5p, miR-1246, miR-5189-5p | MiRNA selection based on microarray data. Normalised to global mean. The combination of miR-451ª and miR-3619-3p showed an AUC of 0.819 |
| **Pan et al.^11^** | 2022 | Discovery  30 CCA  30 Benign  Validation  20 CCA  20 Benign | Yes | miR-141-3p, miR-200a-3p, miR-200b-3p, miR-200c-3p | miRNA selection based on literature. Bile EV miR-200a-3p and miR-200c-3p showed an AUC of 0.88 with a sensitivity of 85% and specificity of 85%. Combined with CA19-9, this improved to an AUC of 0.89. |
| Abbreviations: AUC, area under the curve; CA19-9, carbohydrate antigen 19-9; CCA, cholangiocarcinoma; CP, chronic pancreatitis; EV, extracellular vesicles; PDAC, pancreatic ductal adenocarcinoma; PSC, primary sclerosing cholangitis; ROC, receiver operating characteristic | | | | | |

**References**

1. Shigehara K, Yokomuro S, Ishibashi O, et al. Real-time PCR-based analysis of the human bile microRNAome identifies miR-9 as a potential diagnostic biomarker for biliary tract cancer. *PLoS One*. 2011;6(8):e23584. doi:10.1371/journal.pone.0023584

2. Cote GA, Gore AJ, McElyea SD, Heathers LE, Xu H, Sherman S, Korc M. A pilot study to develop a diagnostic test for pancreatic ductal adenocarcinoma based on differential expression of select miRNA in plasma and bile. *Am J Gastroenterol*. Dec 2014;109(12):1942-52. doi:10.1038/ajg.2014.331

3. Li L, Masica D, Ishida M, et al. Human bile contains microRNA-laden extracellular vesicles that can be used for cholangiocarcinoma diagnosis. *Hepatology*. Sep 2014;60(3):896-907. doi:10.1002/hep.27050

4. Voigtländer T, Gupta SK, Thum S, Fendrich J, Manns MP, Lankisch TO, Thum T. MicroRNAs in Serum and Bile of Patients with Primary Sclerosing Cholangitis and/or Cholangiocarcinoma. *PLoS One*. 2015;10(10):e0139305. doi:10.1371/journal.pone.0139305

5. Le N, Fillinger J, Szanyi S, et al. Analysis of microRNA expression in brush cytology specimens improves the diagnosis of pancreatobiliary cancer. *Pancreatology*. Sep 2019;19(6):873-879. doi:10.1016/j.pan.2019.04.001

6. Han HS, Kim MJ, Han JH, et al. Bile-derived circulating extracellular miR-30d-5p and miR-92a-3p as potential biomarkers for cholangiocarcinoma. *Hepatobiliary Pancreat Dis Int*. Feb 2020;19(1):41-50. doi:10.1016/j.hbpd.2019.10.009

7. Han JY, Ahn KS, Kim YH, Kim TS, Baek WK, Suh SI, Kang KJ. Circulating microRNAs as biomarkers in bile-derived exosomes of cholangiocarcinoma. *Ann Surg Treat Res*. Sep 2021;101(3):140-150. doi:10.4174/astr.2021.101.3.140

8. Ge X, Tang L, Wang Y, et al. The diagnostic value of exosomal miRNAs in human bile of malignant biliary obstructions. *Digestive and Liver Disease*. 2021/06/01/ 2021;53(6):760-765. doi:<https://doi.org/10.1016/j.dld.2020.11.010>

9. Uchihata Y, Arihiro K, Kaneko Y, et al. Analysis of MicroRNA in Bile Cytologic Samples Is Useful for Detection and Diagnosis of Extrahepatic Cholangiocarcinoma. *Am J Clin Pathol*. Jul 1 2022;158(1):122-131. doi:10.1093/ajcp/aqac015

10. Yoshida M, Yukawa H, Hayashi K, et al. Clinical impact of bile-derived exosomal microRNAs as novel diagnostic and prognostic biomarkers for biliary tract cancers. *Cancer Sci*. Jan 2023;114(1):295-305. doi:10.1111/cas.15597

11. Pan Y, Shao S, Sun H, Zhu H, Fang H. Bile-derived exosome noncoding RNAs as potential diagnostic and prognostic biomarkers for cholangiocarcinoma. Original Research. *Frontiers in Oncology*. 2022-August-24 2022;12doi:10.3389/fonc.2022.985089
